# Chrononutrition during Pregnancy and Its Association with Maternal and Offspring Outcomes: A Systematic Review and Meta-Analysis of Ramadan and Non-Ramadan Studies

**DOI:** 10.3390/nu15030756

**Published:** 2023-02-02

**Authors:** Yu-En Chen, See Ling Loy, Ling-Wei Chen

**Affiliations:** 1 Institute of Epidemiology and Preventive Medicine, College of Public Health, National Taiwan University, No. 17 Xu-Zhou Road, Taipei 10055, Taiwan; 2 Department of Reproductive Medicine, KK Women’s and Children’s Hospital, Singapore 229899, Singapore; 3 Duke-NUS Medical School, Singapore 169857, Singapore; 4 Master of Public Health Program, College of Public Health, National Taiwan University, No. 17 Xu-Zhou Road, Taipei 10055, Taiwan

**Keywords:** chrononutrition, meal skipping, meal frequency, night eating, fasting, pregnancy, newborn

## Abstract

Much evidence suggests that food intakes and eating patterns are major determinants of the phase of peripheral circadian clocks, and desynchronization between them is thought to contribute to the development of metabolic disorders. However, much remains to be understood about how different dimensions of chrononutrition during pregnancy affect pregnant women’s and their offspring’s health outcomes. Therefore, we systematically reviewed and integrated all emerging evidence on chrononutrition during pregnancy (including meal skipping, meal frequency, night eating, and (Ramadan) fasting) and their relationships with maternal and offspring outcomes. The results suggest that meal skipping and night eating during pregnancy were generally associated with adverse pregnancy and birth outcomes, whereas no strong conclusion could be reached for meal frequency. In our meta-analysis, Ramadan fasting did not seem to be related with birth weight or gestational age at birth, but evidence for other mother–offspring outcomes was inconsistent. To further elucidate the effect of chrononutrition factors on maternal and offspring health outcomes, larger and well-conducted prospective cohort and interventional studies are needed. In addition, information on covariates such as physical activity, sleep, diet quality and quantity, fasting days, fasting period per day, and trimester exposure should also be collected and considered during analysis.

## 1. Introduction

The nutritional status and dietary intake of women during pregnancy are not only related to their own health, but also the health of their offspring. Since nutritional requirements during pregnancy differ considerably from those of non-pregnant women, the daily reference values of many nutrients and energy intake are different during pregnancy to ensure healthy pregnancy weight gain, optimal fetal development, and favorable long-term outcomes of offspring [1]. Maternal malnutrition and excessive gestational weight gain (GWG) increase the risk of adverse offspring and maternal health outcomes [2]. For example, inadequate GWG was associated with increased risks of preterm delivery and delivering small-for-gestational age infants; excessive GWG was associated with pregnancy-induced hypertension, cesarean delivery, macrosomia [3], and childhood obesity [4]. Hence, optimal dietary intake and nutritional status during pregnancy are vital for maternal and child health.

In addition to dietary content (energy intake and nutrients), multiple lines of evidence now suggest that chrononutrition (interaction between nutrition and the circadian rhythm) can also have a great impact on health [5]. The human circadian rhythm refers to physiological, molecular, and behavioral changes with a cycle length of approximately 24 h. In humans, the “master clock” of circadian rhythm is located in the suprachiasmatic nucleus (SCN) of the anterior hypothalamus, which is primarily regulated by light/dark cycles in order to synchronize the body to the light cycle [6]. In addition, “peripheral clocks” exist in the gut, liver, endocrine organs, adipose tissue, and skeletal muscle, which have their own biological clocks but are also regulated by the SCN [7]. Eating timing, frequency, and regularity are major factors in determining the phase of the peripheral clocks [8,9]. During eating, activation of the insulin-phosphorylated protein kinase B (pPKB)–mammalian target of rapamycin (mTOR) pathway phosphorylates casein kinase 1 (CK1) and glycogen synthase kinase 3 (GSK3), both of which phosphorylate the circadian clock component Period (PER), altering its stability [10]. After hours of fasting, activated 5’AMP-activated protein kinase (AMPK) phosphorylates Crytochromes (CRY) and promotes its degradation [11]. Therefore, frequent eating, the absence of a defined fasting period, and desynchrony between eating and circadian rhythms may disrupt normal metabolic state [12,13].

Ramadan is the ninth month of the Islamic calendar, during which Muslims abstain from all foods and liquids from dawn to sunset [14]. Seasonal and geographical conditions affect the duration of daily fasting, which may vary from 11 to 18 h per day [15]. Sick individuals, pregnant women, and menstruating women are exempt from fasting, but approximately 70–90% of pregnant women still choose to fast [16]. Although Ramadan fasting is not aligned with normal circadian rhythms (fasting during daytime) by disturbing usual eating patterns, there is no conclusive evidence about the impacts of Ramadan fasting during pregnancy on maternal and offspring health [17].

Despite a growing interest and understanding of chrononutrition in the general population, much remains to be understood about how different dimensions of chrononutrition during pregnancy affect health outcomes of pregnant women and their offspring. Therefore, we aimed to systematically review and consolidate all emerging evidence reporting chrononutrition during pregnancy and its relationship with maternal and offspring health outcomes.

## 2. Materials and Methods

### 2.1. Search Strategy

We searched two online databases (PubMed and Embase) from database conception until 19 September 2022 for relevant papers that investigated maternal chrononutrition and mother–offspring outcomes. The search terms were developed according to the PEO (Participants AND Exposure AND Outcome) framework. A combination of the following keywords was used for the search: participants (pregnancy OR gravidity OR gestation OR antenatal OR postpartum OR mother etc.) and exposure (chrononutrition OR meal frequency OR meal skipping OR eating episode etc.). The exact search strategy for the databases is shown in Supplementary Table S1. Since the inclusion of outcome keywords resulted in limited improvement in search specificity and was less comprehensive with regard to a range of health outcomes corresponding to maternal chrononutrition, we left out the outcome keywords.

### 2.2. Study Selection

The identified citations from the online searches were de-duplicated before first-stage screening using titles and abstracts. Full texts of potentially relevant articles were then retrieved and subjected to second-stage screening.

We included original, peer-reviewed, experimental (randomized or non-randomized clinical trials), and observational (prospective/retrospective cohort, case-control, and cross-sectional) studies reporting data on chrononutrition factors during the peri-pregnancy period (pre-pregnancy, pregnancy, and post-delivery) in relation to maternal or child health outcomes.

We excluded (1) meeting abstracts, comments, editorials, study protocols, reviews, and meta-analyses; (2) animal studies and in vitro studies; (3) studies that did not study chrononutrition factors during the peri-pregnancy period in relation to maternal or child outcomes; (4) studies without a control group (e.g., case reports and series); and (5) articles not written in English.

### 2.3. Data Extraction

The data extracted from the included articles included the first author’s name, publication year, study design, city and country of study, population, sample size, study period, duration of fasting per day (for Ramadan studies), personal characteristics of participants (age, BMI), exposure factors, outcomes, gestational age of exposure assessment, and covariates. If the literature did not mention fasting hours during Ramadan (*n* = 20), we estimated the fasting time as the duration from the Islamic Fajr to Maghrib prayers (from dawn to sunset) according to the reported study city and calendar/reported dates of Ramadan fasting.

### 2.4. Risk of Bias Assessment

To assess risk of bias in the included studies, Mixed Method Appraisal Tool Version 2018 (MMAT) was used. The (A) quantitative non-randomized studies criteria used in the MMAT include the following domains: (1) whether the sample is representative of the population; (2) appropriateness of measurement; (3) completeness of results; (4) adjustment of confounders; and (5) compliance with interventions (or in observational studies, whether exposure occurred as intended). The (B) quantitative randomized controlled trials criteria include the following domains: (1) is randomization appropriately performed? (2) Are the groups comparable at baseline? (3) Are there complete outcome data? (4) Are outcome assessors blinded to the intervention provided? (5) Did the participants adhere to the assigned intervention? Within each domain, each study was categorized based on whether they had met the criteria (marked as “Yes”) or not (marked as “No”) (Supplementary Table S2). For the purpose of subgroup analysis in the meta-analysis only, we categorized the studies as having low, medium, or high risk of bias if they did not meet ≤2, 3, or ≥4 (out of 5) of the domain criteria, respectively.

### 2.5. Data Analysis

Due to the mostly heterogenous results from the included studies with various definitions of exposure and outcomes, especially for non-Ramadan studies involving various aspects of maternal chrononutrition factors, most of the results and discussions were narrative (i.e., without meta-analysis). However, since there was a sizeable number of studies involving Ramadan fasting and birth weight/low birth weight and gestational age at birth/preterm birth, we conducted meta-analyses to generate summary estimates. A smaller number of studies also looked at the influence of Ramadan fasting on common maternal outcomes (blood glucose and weight gain), and we pooled these estimates as well.

For meta-analyses, pooled mean differences with 95% confidence intervals were derived for continuous outcomes, whereas pooled odds ratios with 95% confidence intervals were calculated for binary outcomes. A random effects meta-analysis was used in anticipation of heterogeneity due to differences in study design. The Cochran Q test and *I*^2^ statistic were used to evaluate statistical heterogeneity among studies, and *I*^2^ values of 25%, 50%, and 75% correspond to low, moderate, and high degrees of heterogeneity, respectively [18]. For outcomes with a sufficient number of studies, we conducted stratified analyses to identify potential sources of heterogeneity by different study-level characteristics (number of participants, age, duration of fasting per day, region, BMI, and MMAT). If studies presented data grouped by fasting days or duration of fasting per day, we selected the group with the longest fasting duration (i.e., most extreme contrast compared to the non-fasting group). If a study provided effect estimates by trimester, we derived a within-study pooled mean and standard deviation first before including these data in the meta-analysis. However, for the weight gain outcome, because almost all studies assessed the influence of Ramadan fasting during second trimester, we only included data from the second trimester in *Kiziltan* et al. study [19]. All analyses were performed using STATA version 16 (StataCorp, College Station, TX, USA).

## 3. Results

### 3.1. Search Results

**Figure 1:**
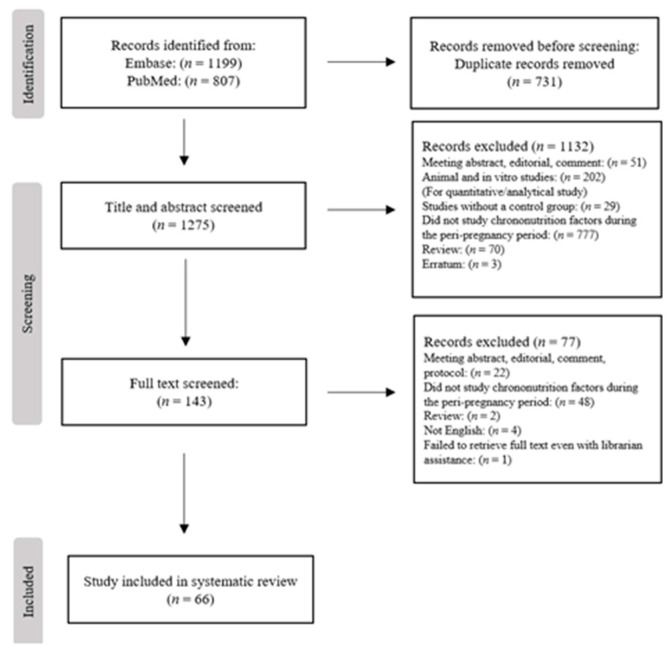
Flowchart for study selection.

In total, 2006 articles were identified from online databases (PubMed and Embase) in the initial search. After excluding duplicates (*n =* 731), 1275 articles were screened based on title and abstract, of which 1132 were excluded. Full texts of the remaining 143 articles were further screened, and we finally included 66 articles in our systematic review (41 Ramadan and 25 non-Ramadan). The detailed study selection process is shown in Figure 1.

### 3.2. Study Characteristics

The included literature investigated the relationships of chrononutrition during pregnancy with maternal, fetal, neonatal, and childhood outcomes. The chrononutrition factors assessed can be broadly categorized into meal skipping (*n* = 7), night eating (*n* = 8), meal frequency (*n* = 11), night fasting duration (*n* = 2), and Ramadan fasting (*n* = 41). As mentioned above, due to substantially heterogenous exposure and outcomes, the non-Ramadan fasting literature on meal frequency, meal skipping, night eating, and night fasting duration were reviewed qualitatively. For the Ramadan fasting literature, some important birth outcomes [gestational age at birth (*n* = 11); risk of preterm birth (*n* = 7); birth weight (*n* = 19); and risk of low birth weight (*n* = 4)] and maternal outcomes [weight gain (*n* = 8) and blood sugar (*n* = 5)] were analyzed quantitatively, while other outcomes were less frequently reported and thus reviewed qualitatively.

### 3.3. Maternal Chrononutrition Factors and Maternal and Child Outcomes: Non-Ramadan Studies

#### 3.3.1. Meal Skipping

**Figure 2:**
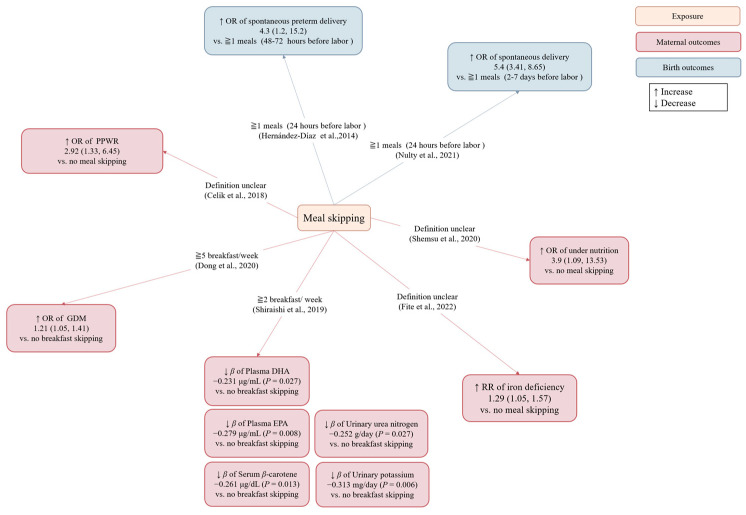
Associations of meal skipping with maternal and birth outcomes. Regression coefficient: beta estimates (*β*); docosahexaenoic acid (DHA); eicosapentaenoic acid (EPA); gestational diabetes mellitus (GDM); odds ratio (OR); relative risk (RR); postpartum weight retention (PPWR) [20,21,22,23,24,25,26].

Studies investigating meal skipping during pregnancy and maternal/offspring outcomes are summarized in Figure 2 and Table 1.

In two self-matched case-crossover studies (used to assess health event triggers) from the United States, compared with a week [20] or three days [21] before labor (control period), the odds of imminent spontaneous labor were 5.4 (95% CI: 3.41, 8.65) [20] and 4.3 (95% CI: 1.2, 15.2) [21] times as high within 24 h (hazard period) of skipping one or more meals [20,21]. However, these results may be affected by reverse causality since irregular meal intake can be a sign rather than a cause of the approaching labor due to a lack of appetite [20].

Three articles examined the association of meal skipping with maternal nutritional status. Two cross-sectional studies from Ethiopia showed that skipping meals during pregnancy was positively associated with under-nutrition (OR = 3.9, 95% CI: 1.09, 13.53) [22] and iron deficiency anemia [23] (RR = 1.29; 95% CI: 1.05, 1.57). The third cross-sectional study from Japan showed that healthy pregnant women who skipped breakfast two or more times per week (*n* = 37) had lower intakes of protein after adjusting for energy intakes than those who did not skip breakfast (*n* = 60) (*p* = 0.019). In addition, they also had lower plasma docosahexaenoic acid (DHA)(*p* = 0.027), plasma eicosapentaenoic acid (EPA)(*p* = 0.008), serum *β*-carotene (*p* = 0.013), urinary urea nitrogen (*p* = 0.027), and urinary potassium (*p* = 0.006) compared to non-breakfast skippers [24].

Furthermore, another study showed that compared with daily breakfast eaters, healthy Japanese pregnant women who skipped breakfast more frequently had a higher odds of developing gestational diabetes (GDM) [OR (95% CI): 1.09 (0.93, 1.27), 1.14 (0.96, 1.34), and 1.21 (1.05, 1.41) for 1–2 times/wk, 3–4 times/wk, and 5–7 times/wk breakfast skipping, respectively] [25]. In addition, a cross-sectional study found that Turkish mothers who skipped meals during pregnancy were 2.92 (95% CI: 1.33, 6.45) times more likely to maintain weight at 12–18 months postpartum than those who did not skip meals [26]. 

In summary, these results indicate that skipping meals during pregnancy may be related to maternal malnutrition [22,23,24], GDM [25], and weight status [26]. However, since most studies are cross-sectional in nature, it is unclear whether these associations are causal.

**Table 1:** Characteristics of studies on meal skipping in relation to maternal and birth outcomes.

Author (Country, Year of Publication)	Study Design	Population	Number of Participants	Age, y (mean)	BMI, kg/m^2^	Period of Exposure Assessment	Comparison of Exposure	Outcomes	Covariates	Main Findings
Nulty (United States, 2021) [20]	Case-crossover study	Pregnant women with spontaneous labor	607	29.1	24.4 (Pre-pregnancy)	One week before delivery	Meal skipping ≥1 times/day within 24 h of labor vs. the week before labor	Spontaneous delivery	Na	Meal skipping later in pregnancy was associated with an increased likelihood of imminent spontaneous labor.
Hernández-Día (United States, 2014) [21]	Case-crossover study	Pregnant women with premature labor or preterm premature rupture of membranes	100	31.5	25.6 (Pre-pregnancy)	0–72 h before preterm delivery	Meal skipping ≥1 times/day within 24 h of labor vs. during the 48–72 h before labor	Spontaneous preterm labor and preterm premature rupture of membranes	Na	Meal skipping was associated with an increased risk for spontaneous preterm labor and preterm premature rupture of membranes within the subsequent 24 h.
Shemsu (Ethiopia, 2020) [22]	Cross-sectional study	Pregnant women	378	28.9	na	(Definition unclear)	Meal skipping vs. no meal skipping	Under-nutrition (MUAC < 21.0 cm)	Wealth data	Meal skipping was associated with an increased odds of under-nutrition.
Fite (Ethiopia, 2022) [23]	Cross-sectional study	Pregnant women	446	24.8	na	1–40 weeks of gestation	Meal skipping vs. no meal skipping	Iron deficiency (serum ferritin <15 μg/L)	Inflammation	Meal skipping was associated with an increased risk of iron deficiency.
Shiraishi (Japan, 2019) [24]	Cross-sectional study	Healthy pregnant women	97	35.1	20.4 (Pre-pregnancy)	15–18 weeks of gestation	Breakfast skipping ≥2 times/week vs. no breakfast skipping	Circulating and urinary levels of nutrients	Educational levels, supplement use, LDL-C (only for serum vitamin E levels)	Breakfast skipping ≥2 times/week was associated with lower plasma EPA, plasma DHA, serum *β*-carotene, urinary urea nitrogen, and urinary potassium compared to no breakfast skipping
Dong (Japan, 2020) [25]	Cohort study	Healthy pregnant women	84,669	30.7	21.1 (Pre-pregnancy)	26–28 weeks of gestation	Breakfast skipping 1–2, 3–4, or 5–7 times/week vs. no breakfast skipping	GDM	Age, smoking status, drinking status, education level, occupation, household income, history of depression, history of having infants with macrosomia, history of polycystic ovarian syndrome, marital status, parity, physical activity, total daily energy intakes, Western dietary pattern scores, BMI	Breakfast skipping 5–7 times/week before and during early pregnancy was associated with an increased odds of developing GDM compared to no breakfast skipping.
Celik (Turkey, 2018) [26]	Cross-sectional study	The mothers were in the 12–18 month postpartum, and gave birth between the 37th and 42nd weeks.	239	30.8	22.6 (Pre-pregnancy)	(Definition unclear)	Meal skipping vs. no meal skipping	PPWR (at 12–18 months)	na	Meal skipping was associated with higher PPWR.

Body mass index (BMI); docosahexaenoic acid (DHA); eicosapentaenoic acid (EPA); gestational diabetes mellitus (GDM); low density lipoprotein-cholesterol (LDL-C); mid-upper arm circumference (MUAC); postpartum weight retention (PPWR).

#### 3.3.2. Night Eating

**Figure 3:**
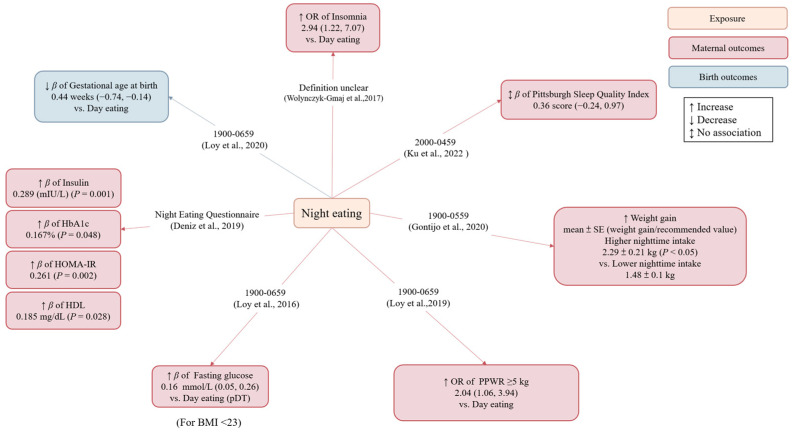
Associations of night eating with maternal and birth outcomes. Regression coefficient: beta estimates (*β*); hemoglobin A1c (HbA1c); high density lipoprotein (HDL) cholesterol; homeostasis model assessment for insulin resistance (HOMA-IR); odds ratio (OR); predominantly daytime (pDT); postpartum weight retention (PPWR); relative risk (RR). Due to a different approach used, the study by Messika et al. was not included in the figure; please refer to main text for the results [27,28,29,30,31,32,33].

Studies investigating night eating during pregnancy and its associations with maternal/offspring outcomes are summarized in Figure 3 and Table 2.

A prospective cohort study in Singapore showed that maternal night eating (defined as consuming >50% of total daily energy intake during 1900–0659 h) at 26–28 weeks gestation was associated with a 0.44-week reduction in gestational age at birth (95% CI: −0.74, −0.14), but its association with preterm birth did not reach statistical significance (OR = 2.14, 95% CI: 0.99, 4.66) [27].

Two cross-sectional studies investigated the relationship between maternal night eating and sleep quality. One study from Poland found that eating at night (definition unclear) at 28–41 weeks gestation was associated with insomnia (defined as an Athens Insomnia Scale (AIS) score >8) (OR = 2.94, 95% CI: 1.22, 7.07) [28]. Another study from Singapore assessed sleep quality using the Pittsburgh Sleep Quality Index (PSQI) questionnaire and found that night eating (defined as eating during 2000-0459 h) at 18–24 weeks gestation was associated with higher PSQI scores (reflecting poorer sleep) (*β =* 0.68, 95% CI: 0.03, 1.32). However, further adjustment for negative emotions, lifestyle behaviors, maternal age, etc. attenuated the association between night eating and sleep quality (*β =* 0.36, 95% CI: −0.24, 0.97) [29].

Two studies explored the associations of maternal night eating with GWG and postpartum weight retention (PPWR). A prospective cohort study from Brazil collected three 24 h dietary recalls in each trimester and pregnant women were classified as having a “lower” or “higher” night eating pattern if caloric consumption during 1900-0559 h was below or above the median of the population, respectively, for at least two trimesters. The results showed that women in the higher night eating group had lower energy intakes (*p* = 0.009) but gained more weight than recommended in the third trimester compared to the lower night eating group (*p* < 0.05) [30]. Another study from Singapore defined night eating as consuming >50% of total daily energy intake during 1900–0659 h in pregnant women, which was assessed using a 24 h dietary recall at 26–28 weeks gestation. After confounder adjustment, night eating at 26–28 weeks gestation was associated with higher odds of substantial PPWR ≥5 kg at 18 months (OR = 2.04, 95% CI: 1.06, 3.94) [31].

There were two studies that investigated metabolic consequences of night eating. The first prospective cohort study from Singapore divided pregnant women into overweight (≥23 kg/m^2^) and lean (<23 kg/m^2^) groups and then classified them as being predominantly daytime (pDT) or predominantly nighttime (pNT) eaters based on whether they consumed a greater percentage of calories from 0700 to 1859 h or from 1900 to 0659 h. The results showed that pNT eaters had lower energy intakes at 26–28 weeks gestation in the lean group (*p* = 0.019) but not in the overweight group. However, after adjusting for confounders, pNT feeding was associated with a higher fasting glucose level in the lean group (*β =* 0.16 mmol/L, 95%CI: 0.05, 0.26); no association was noted for postprandial plasma glucose outcome [32]. Another cross-sectional study from Turkey reported that a higher total score from the Night Eating Questionnaire was positively associated with hemoglobin A1C (HbA1c), insulin resistance, insulin, and high density lipoprotein (HDL) cholesterol levels at 28–38 weeks gestation (*p* < 0.05) [33]. These observational findings were supported by a recent randomized controlled trial involving 103 Israeli women with GDM who were assigned to either the chrononutritional and sleep hygiene intervention group (*n* = 33) or the control group (*n* = 70) at 25–29 weeks gestation [34]. The chrononutritional recommendations for the intervention group from 25–29 weeks gestation to delivery were as follows: (1) reduce carbohydrate intake by 10 to 15% during the evening interval (1800–0600); (2) eat breakfast up to 30 min from waking up and eat dinner within and up to 12 h from waking up; and (3) avoid eating 1.5 h before going to sleep. The trial showed a significant reduction of suboptimal glycemic control (defined by <80% of the plasma glucose values at target) in the intervention group compared to the control group (RR = 0.28, 95% CI: 0.18, 0.81). However, the intervention had no effect on birth weight and gestational age at birth [34].

Overall, eating at night during pregnancy seems to be associated with higher preterm birth risk, impaired glucose metabolism, poor sleep quality, higher GWG, and higher risk of PPWR. These adverse complications may be related to disrupted circadian rhythms by night eating [35].

**Table 2:** Characteristics of studies on night eating in relation to maternal and birth outcomes.

Authors, Publication Date	Study Design	Population	Number of Participants	Participants’ Age, y	BMI, kg/m^2^	Period of Exposure Assessment	Dietary Assessment Methods	Exposure	Outcomes	Covariates	Main Findings
Loy (Singapore, 2020) [27]	Cohort study	Healthy pregnant women	673	30.9	<23: 53.2% ≥23: 46.8%	26–28 weeks of gestation	24 h dietary recall	Night eating (defined as consuming >50% of total daily energy intake during 1900–0659 h) vs. day eating	(1) Gestational age at birth (2) Preterm birth	Age, ethnicity, education, monthly household income, employment status, night shift, physical activity, early pregnancy BMI, anxiety score, eating episodes, total daily energy intake, infant sex, bedtime, GDM	Night eating was associated with shorter gestation length, but its association with preterm birth did not reach statistical significance.
Wolynczyk-Gmaj (Poland, 2017) [28]	Cross-sectional study	Pregnant women	202	30.6	26.9 (third trimester of pregnancy)	28–41 weeks of gestation	Harvard Light Exposure Assessment questionnaire	Night eating (definition unclear) vs. day eating	Sleep quality (insomnia)	Tingling in the legs, nightmares, snoring, myoclonus, higher depression scores, higher hyperarousal	Night eating was associated with an increased odds of insomnia (defined as an Athens Insomnia Scale (AIS) score >8).
Ku (Singapore, 2022) [29]	Cross-sectional study	Healthy pregnant women	299	31.09	22.89 (pre-pregnancy)	18–24 weeks of gestation	Questionnaire	Night eating (defined as eating during 2000-0459 h)	Sleep quality	Education, employment status, working overtime, pre-pregnancy BMI, negative emotion	Night eating was associated with higher PSQI scores (reflecting poorer sleep). Further adjustment for negative emotions, lifestyle behaviors, maternal age, etc. attenuated the association between night eating and sleep quality
Gontijo (Brazil, 2020) [30]	Cohort study	Healthy pregnant women	100	27.7	24.25	The first/second/third trimester	24 h dietary recall	Higher night eating vs. lower night eating [caloric consumption during 1900-0559 h was above (higher night eating) or below (lower night eating) the median of the population]	GWG	Age, pre-pregnancy BMI, education, chronotype, physical activity, frequency of nausea	Women in the higher night eating group gained more weight (weight gain/recommended value) in the third trimester compared to the lower night eating group
Loy (Singapore, 2019) [31]	Cohort study	Pregnant women	687	31.3	23.6 (≤14 weeks gestation)	26–28 weeks of gestation	24 h dietary recall	Night eating (consuming >50% of total daily energy intake during 1900–0659 h) vs. day eating	PPWR (≥5 kg at 18 months)	Age, ethnicity, education, parity, night shift, total Edinburgh Postnatal Depression Scale score, total daily energy intake, BMI, bedtime, GDM, GWG, feeding in the first six months	Night eating was associated with higher odds of substantial PPWR
Loy (Singapore, 2016) [32]	Cohort study	Healthy pregnant women	985	30.7	<23: 54.2% ≥23: 45.8%	26–28 weeks of gestation	24 h dietary recall	pNT vs. pDT [consuming a greater percentage of calories from 0700 to 1859 (pDT) hours or from 1900 to 0659 h (pNT)]	Blood glucose	Age, education, ethnicity, physical activity, sleep duration, total daily energy intake	Night eating was associated with higher fasting glycemia in lean but not in overweight women.
Deniz (Turkey, 2019) [33]	Cross-sectional study	Healthy pregnant women	148	28.9	30.66	28–38 weeks of gestation	Night Eating Questionnaire	Night eating	(1) Blood glucose (2) Blood lipid (3) Birth weight	na	Night eating was associated with higher HbA1c, insulin resistance, insulin, and HDL.
Messika (Israel, 2022) [34]	Randomized controlled trial	Pregnant women with GDM	103	33.6	27.7 (pre-pregnancy)	Intervention and follow up from gestational week 25–29 until delivery	24 h dietary recall	Intervention group: chrononutrition and sleep hygiene program vs. control group: usual GDM care	(1) Glycemic control (2) Birth weight (3) Gestational age at birth	Age, pre-pregnancy BMI, number of children, history of GDM, LGA birth	A significant reduction in suboptimal glycemic control in the intervention compared to control group. The intervention had no effect on birth weight or gestational age at birth.

Body mass index (BMI); gestational diabetes mellitus (GDM); gestational weight gain (GWG); high density lipoprotein (HDL); hemoglobin A1C (HbA1C); large for gestational (LGA); postpartum weight retention (PPWR); predominantly daytime (pDT); predominantly nighttime (pNT).

#### 3.3.3. Meal Frequency

**Figure 4:**
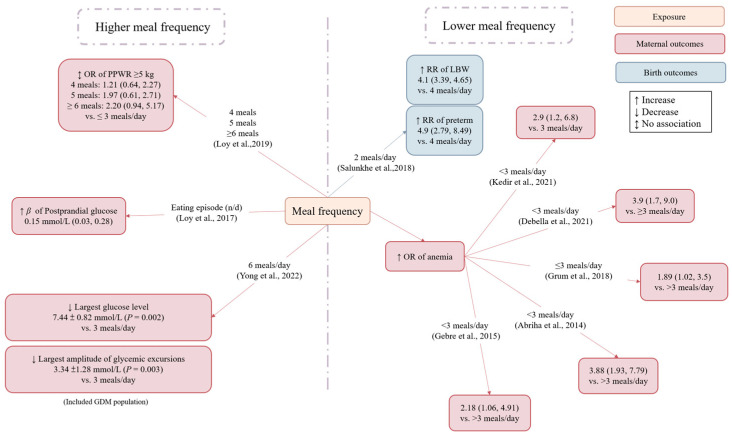
Associations of meal frequency with pregnancy and birth outcomes. Regression coefficient: beta estimates (β); gestational diabetes mellitus (GDM); low birth weight (LBW); postpartum weight retention (PPWR); odds ratio (OR); relative risk (RR). Due to different approaches used, the studies by Englund-Ögge et al. and Ainscough et al. were not included in the figure; please refer to main text for the results [31,36,39,40,41,42,43,44,45].

Studies investigating meal frequency during pregnancy and its associations with maternal/offspring outcomes are summarized in Figure 4 and Table 3.

Three cohort studies investigated the relationship between maternal meal frequency and neonatal outcome. Salunkhe et al. assessed the meal frequency of 380 Indian pregnant women at 14–26 weeks gestation using a 24 h dietary recall and found that participants who consumed four or five meals a day had higher energy intakes than those having ≤3 meals per day (*p* < 0.001). In addition, participants consuming two and three meals a day had a higher risk of delivering low birth weight (RR = 4.1, 95% CI: 3.39, 4.65 for two meals and RR = 11.9, 95% CI: 6.8, 20.9 for three meals) and preterm birth (RR = 4.9, 95% CI: 2.79, 8.49 for two meals and RR = 3.1, CI: 1.8, 5.4 for three meals) infants as compared to participants having ≥4 meals per day [36]. Using principal component factor analysis, Englund-Ögge et al. derived several “meal frequency patterns”, namely “snack pattern”, “main meal pattern”, and “dinner pattern” among 65,487 Swedish pregnant women; greater adherence to the “main meal pattern” (implying regular intakes of breakfast, lunch, and dinner) was associated with a reduced risk of preterm birth [hazard ratio (95% CI): 0.89 (0.80, 0.98) and 0.90 (0.81, 0.99) for the third and fourth quartiles of “main meal pattern” score, respectively] [37]. Ainscough et al. recruited only pregnant women with overweight and obesity from Ireland and identified three mutually exclusive meal frequency pattern categories [“main meal pattern” (3 main meals + 0–3 snacks), “large meal pattern” (≤2 main meals + <2 snacks), and “snack pattern” (3 main meals + >3 snacks or ≤2 main + ≥2 snacks)] based on preferences for consuming main meals or snacks. There were no differences in nutrient and energy intakes across the meal patterns at 16 and 28 weeks gestation. However, pregnant women with a large meal pattern at 28 weeks gestation were more likely to deliver macrosomic babies compared to those with main meal and snack patterns (*p* = 0.008 based on a chi-squared test) [38].

Two cohort studies explored the association of meals pattern during pregnancy with GWG and PPWR. Ainscough et al. showed that term GWG was lower in women with the aforementioned large meal pattern than those with the main meal or snack patterns at 16 weeks gestation [38]. Another cohort study from Singapore assessed the relationship between eating episodes at 26–28 weeks gestation and PPWR. No associations were observed between eating episodes and PPWR (weight at 18 months postpartum first antennal visit weight ≥5 kg) [31].

Two studies investigated the association between eating frequency and blood glucose regulation in pregnant women. One cross-sectional study included 1061 Singapore pregnant women and used 24 h dietary recalls to determine eating episodes. The results showed that women with more frequent eating episodes had greater total energy intakes (*p* < 0.001), and each additional daily eating episode was associated with 0.15 mmol/L higher 2 h glucose (95% CI: 0.03, 0.28) but not with fasting glucose [39]. Another randomized crossover trial with isocaloric meals provided to 10 women with GDM showed that increasing meal frequency to six meals a day for one day, as compared with three meals a day, could significantly affect glycemic excursions measured by continuous glucose monitoring. Specifically, higher meal frequency reduced the peak glucose level, standard deviation, coefficient variation, mean amplitude, and the largest amplitude of glycemic excursions (all *p* < 0.05), but no differences were observed for the mean and lowest glucose levels [40].

Five cross-sectional studies, all conducted in Ethiopia, found that eating fewer than three meals a day increased the risk of anemia in pregnant women by 1.9–3.9 times as compared with eating more than three meals a day [41,42,43,44,45].

In summary, studies on the associations of meal frequency during pregnancy with maternal and child outcomes provided mixed findings. Higher meal frequency may be associated with lower risk of low birth weight, preterm birth [36], anemia [41,42,43,44,45], and less glycemic variability [40], but it may have the potential to increase postprandial gestational glycemia [39]. More studies are needed to determine the optimal meal frequency for pregnant women.

**Table 3:** Characteristics of studies on meal frequency in relation to maternal and birth outcomes.

Author (Country, Year of Publication)	Study Design	Population	Number of Participants	Participants’ Age, y	BMI, kg/m^2^	Period of Exposure Assessment	Comparison of Exposure	Outcomes	Covariates	Main Findings
Salunkhe (India, 2018) [36]	Cohort study	Healthy pregnant women	380	na	na	14–26 weeks of gestation (the second trimester)	2 or 3 meals/day vs. ≥4 meals/day	(1) Birth weight (2) Gestational age at birth (3) Low birth weight (4) Preterm birth	na	Increasing meal frequency from two to four meals was associated with higher birth weight, longer gestational age at birth, and a reduced risk of low birth weight and preterm birth delivery.
Englund (Ögge (Sweden, 2017) [37]	Cohort study	Healthy pregnant women	65,487	<35y: 83.2% ≥35y: 16.8%	<18.5: 3.3% 18.5–24.9: 66% 25–29.9: 20.2% ≥30: 8%	na	The second, third, and fourth quartiles of meal patterns [snack meal, main meal (breakfast, lunch, and dinner), evening meal] vs. the first quartile of each meal pattern	Preterm birth	Age, pre-pregnancy BMI, height, parity, total daily energy intake, education, marital status, smoking, income, history of preterm birth, other meal frequency patterns	Regular consumption of main meals (breakfast, lunch, and dinner) was associated with a lower preterm birth risk.
Ainscough (Ireland, 2020) [38]	Cohort study	Pregnant women with overweight and obesity	143	32.1	29.1	16–28 weeks of gestation	Meals pattern [main meal pattern (3 main meals + 0–3 snacks), large meal pattern (≤2 main meals + <2 snacks), and snack pattern (3 main meals + >3 snacks or ≤2 main meals + ≥2 snacks)]	(1) Blood glucose (2) GWG (3) Gestational age at birth (4) Macrosomia	na	Women with a large meal pattern at 16 weeks gestation had lower GWG than those with the main meal or snack pattern. Women with a large meal pattern at 28 weeks gestation were more likely to give birth to a macrosomic infant than those with the main meal or snack pattern. Meal patterns were not associated with blood glucose or gestational age at birth.
Loy (Singapore, 2017) [39]	Cross-sectional study	Healthy pregnant women	1061	30.7	23.6 (≤14 weeks gestation)	26–28 weeks of gestation	Eating episodes (n/day)	Blood glucose	Age, ethnicity, education, employment status, night shift status, parity, BMI, physical activity, sleep duration, bedtime, total daily energy intake, % energy during nighttime, % energy from protein, % energy from fat	Each additional daily eating episode was associated with higher 2 h glucose but not with fasting glucose.
Yong (China, 2022) [40]	Randomized crossover study	Pregnant women with GDM	10	30.1	30.1	26.7 weeks (Average)	(1) Three meals a day (2) Six meals a day	Blood glucose	na	Increasing the number of meals decreased the standard deviation and mean amplitude of glycemic excursions, the largest amplitude of glycemic excursions, coefficient variation, and peak glucose level but not the mean glucose level or the lowest glucose level
Loy (Singapore, 2019) [31]	Cohort study	Pregnant women	687	31.3	23.6 (≤14 weeks gestation)	26–28 weeks of gestation	≥6 or 5 or 4 meals/day vs. ≤3 meals/day	PPWR (≥5 kg at 18 months)	Age, ethnicity, education, parity, night shift, total Edinburgh Postnatal Depression Scale score, total daily energy intake, BMI, bedtime, GDM, GWG, feeding in the first six months	Eating episodes were not associated with 18-month PPWR.
Kedir (Ethiopia, 2021) [41]	Cross-sectional study	Pregnant women	284	33.1% 18–22 y 53.5% 23–34 y 13.4% ≥ 35 y	na	1–40 weeks of gestation (the first to third trimester)	<3 meals/day vs. 3 meals/day	Anemia	A history of heavy menstrual bleeding, the lack of animal-origin food at least once a week, short birth interval, education	Eating <3 meals a day increased anemia risk.
Debella (Ethiopia, 2021) [42]	Cross-sectional study	Healthy pregnant women	405	26.6	na	(Definition unclear)	<3 meals/day vs. ≥3 meals/day	Anemia	Place of residence, marital status, ANC visit, birth interval, history of contraceptive use, IFA supplementation, blood loss in the current pregnancy, drinking alcohol, eating leafy vegetables, drinking milk with tea after meals	Eating <3 meals a day increased anemia risk.
Grum (Ethiopia, 2018) [43]	Cross-sectional study	Pregnant women	638	27	na	(Definition unclear)	≤3 meals/day vs. >3 meals/day	Anemia	Education, birth interval, malaria attack in last one year, excessive menstrual bleeding, pregnancy-related complication	Eating ≤ 3 meals a day increased anemia risk.
Abriha (Ethiopia, 2014) [44]	Cross-sectional study	Healthy pregnant women	619	27.4	na	(Definition unclear)	3 or <3 meals/day vs. >3 meals/day	Anemia	Age category, family monthly income, marital status, occupational status	Eating <3 meals a day increased anemia risk.
Gebre (Ethiopia, 2015) [45]	Cross-sectional study	Healthy pregnant women	714	25.8	≤20: 21.8% 20–24.9: 20.9% ≥25: 57.3%	26.7 (Average)	3 or < 3 meals/day vs. >3 meals/day	Anemia	Marital status, residence, education, family monthly income, number of visits, age of the women at first marriage, BMI, iron supplementation, nutrition education	Eating <3 meals a day increased anemia risk.

Antenatal care (ANC); body mass index (BMI); gestational diabetes mellitus (GDM); gestational weight gain (GWG); iron folic acid (IFA); postpartum weight retention (PPWR).

#### 3.3.4. Non-Ramadan (Night) Fasting Duration

In a cross-sectional analysis within a Singapore prospective cohort, women with longer night-fasting intervals had lower total energy intakes (*p* < 0.001), and each hourly increase in night fasting interval during 26–28 weeks gestation was associated with a 0.03 mmol/L lower fasting glucose (95% CI: −0.06, −0.01 mmol/L). However, no association was noted for 2 h postprandial blood glucose [39]. In the same cohort, there was no consistent association between higher night fasting duration during pregnancy and substantial PPWR (≥5 kg) at 18 months [31]. Given the dearth of research investigating health impacts of habitual fasting duration during pregnancy, more studies in this area are warranted.

### 3.4. Ramadan Fasting

Studies investigating Ramadan fasting during pregnancy and its associations with maternal/offspring outcomes are summarized in Supplementary Table S3.

#### 3.4.1. Maternal Outcomes: Quantitative Analyses

##### Weight Gain

**Figure 5:**
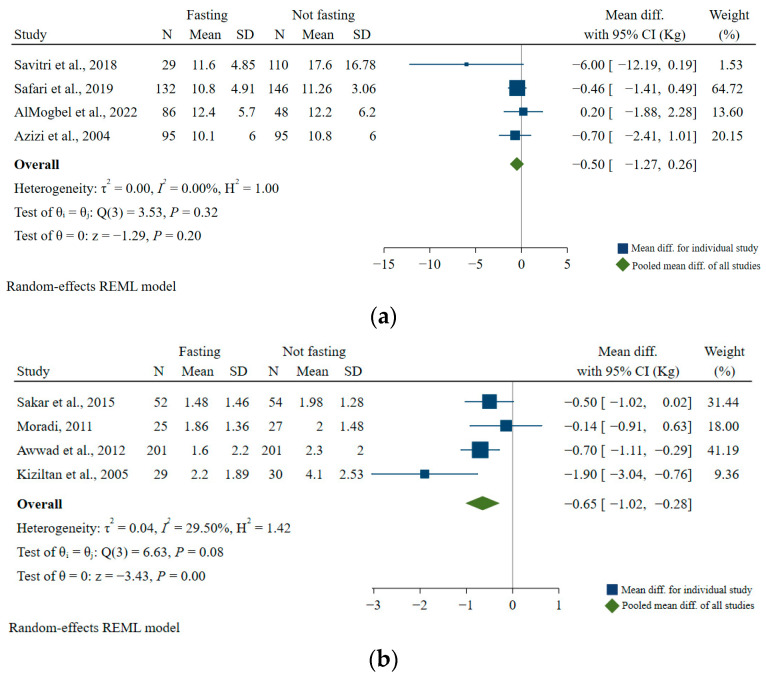
Association of Ramadan fasting with (**a**) weight gain throughout pregnancy and (**b**) weight gain during Ramadan [19,46,47,48,49,50,51,52].

Four studies examined the association between Ramadan fasting and GWG throughout pregnancy. Two of the studies showed lower GWG in the Ramadan fasting group compared with the non-fasting group [46,47], but the other two studies reported no difference between the two groups [48,49]. In our meta-analysis, GWG was not significantly different (pooled mean difference (MD): 0.5 kg; 95% CI: −1.27, 0.26; *I*^2^ = 0.00%) in the Ramadan fasting group compared with the non-fasting group (Figure 5a). In addition, there were four studies that investigated the association between Ramadan fasting and GWG during Ramadan. Two of the studies showed that the Ramadan fasting group had a lower mean GWG during Ramadan than the non-fasting group [19,50], but the other two studies showed no differences between the groups [51,52]. In our meta-analysis, GWG during Ramadan was significantly lower in the fasting group compared with the non-fasting group (pooled MD: 0.65 kg; 95% CI: −1.02, −0.28; *I*^2^ = 29.5%) (Figure 5b).

##### Fasting Blood Glucose

**Figure 6:**
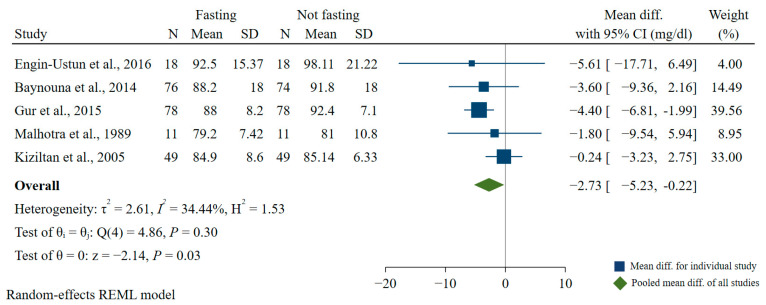
Association of Ramadan fasting with fasting blood glucose [19,53,54,55,56].

Five studies explored the association of Ramadan fasting during pregnancy with fasting blood glucose [19,53,54,55,56]. Results of our meta-analysis showed that fasting during Ramadan significantly reduced fasting blood glucose by 2.73 mg/dl (95% CI: −5.23, −0.22) compared with the non-fasting group (*I*^2^ = 34.44%) (Figure 6).

#### 3.4.2. Birth Outcomes: Quantitative Analyses

##### Gestational Age at Birth

**Figure 7:**
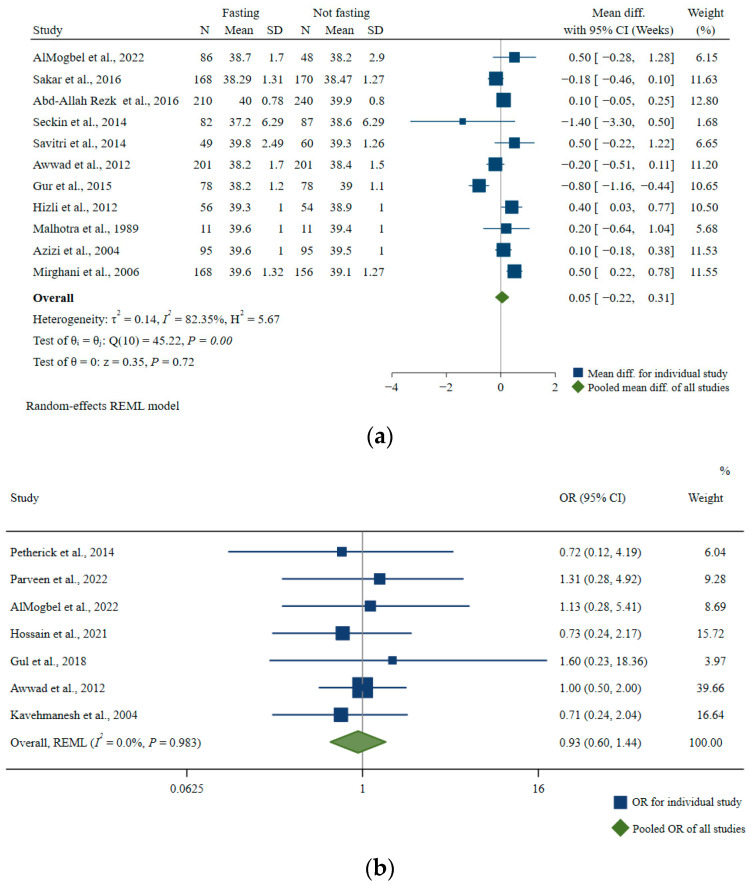
Association of Ramadan fasting with (**a**) gestational age at birth and (**b**) odds of preterm birth [48,49,50,56,57,58,59,60,61,62,63,64,65,66,67].

Eleven articles examined the association of Ramadan fasting with gestational age at birth [48,49,50,55,56,57,58,59,60,61,62]. The pooled mean difference between fasting and non-fasting groups was not statistically significant (pooled MD: 0.05, 95% CI: −0.22, 0.31, *I*^2^ = 82.35%) (Figure 7a). In subgroup analyses according to number of participants, age, duration of fasting per day, region, BMI, and risk of bias, the heterogeneity remained high (*I*^2^ = 63.89–84.14%) (Table 4).

In addition, seven studies investigated the relationship between Ramadan fasting and the risk of preterm birth (gestational age at birth <37 completed weeks) [48,50,63,64,65,66,67]. Compared with the non-fasting group, the pooled OR of preterm birth in the Ramadan fasting group was 0.93 (95% CI: 0.60, 1.44; *I*^2^ = 0.0%) (Figure 7b).

**Table 4:** Subgroup analysis of Ramadan fasting vs. gestational age at birth relationship.

Characteristics	Number of Studies	Pooled Mean Difference, Week	95% CI, Week	*I* ^2^	*p*-For Heterogeneity
**All studies**	11	0.05	−0.22, 0.31	82.35	<0.01
**Number of participants ^a^**					
≤219	7	0.04	−0.39, 0.47	77.74	<0.01
>219	4	0.06	−0.25, 0.37	84.14	<0.01
**Age (years old) ^a^**					
≤27.2	8	−0.03	−0.35, 0.29	82.54	<0.01
>27.2	3	0.23	−0.26, 0.72	80.39	<0.01
**Duration of fasting per day (hours) ^a^**					
≤15.5	7	0.21	0.00, 0.42	63.89	0.02
>15.5	4	−0.40	−0.90, 0.11	70.40	0.02
**Region**					
Middle East/Northeast Africa	4	0.13	−0.13, 0.39	77.74	0.01
Oceania/Europe	7	−0.02	−0.45, 0.42	78.60	<0.01
**BMI (kg/m^2^)**					
<25	2	−0.49	−1.08, 0.09	83.46	0.01
≥25	3	0.17	−0.28, 0.63	70.29	0.03
**MMAT (risk of bias)**					
Low	7	−0.08	−0.48, 0.33	75.90	<0.01
Medium/High	4	0.19	−0.10, 0.49	81.29	<0.01

^a^ Cutoffs were based on median values of the included studies. Body mass index (BMI); Mixed Method Appraisal Tool Version 2018 (MMAT).

##### Birth Weight

**Figure 8:**
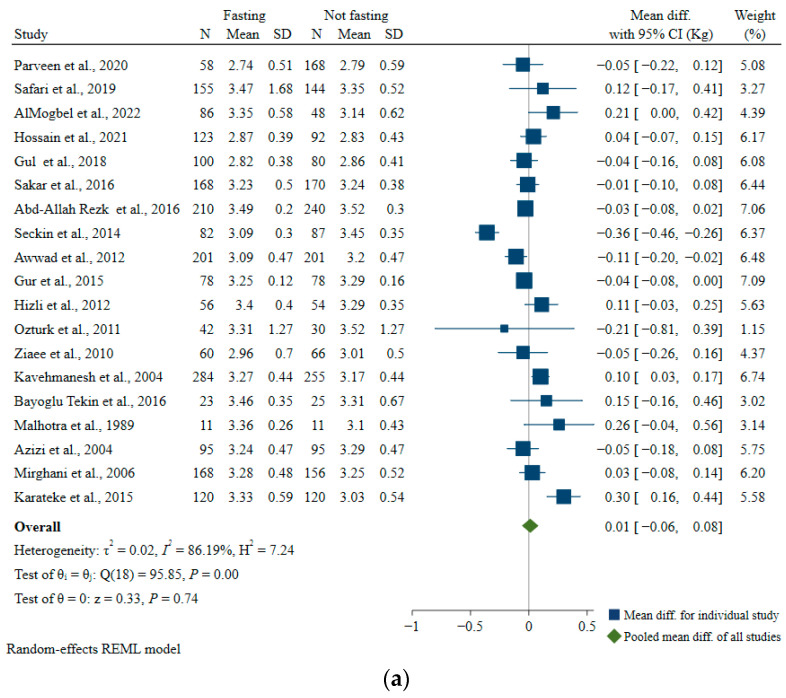

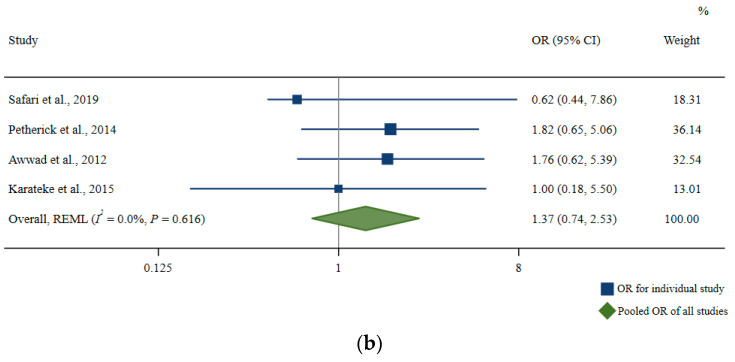
Associations of Ramadan fasting with (**a**) birth weight and (**b**) odds of low birth weight [47,48,49,50,55,56,57,58,59,61,62,63,64,65,66,67,68,69,70,71].

A total of 19 included articles analyzed the relationship between maternal fasting during Ramadan and birth weight [47,48,49,50,55,56,57,58,59,61,62,64,65,66,67,68,69,70,71]. The pooled mean difference between fasting and non-fasting groups was not statistically significant (pooled MD: 0.01 kg, 95% CI: −0.06, 0.08; *I*^2^ = 86.19%) (Figure 8a). Heterogeneity remained mostly high in subgroups, with exceptions noted for studies from South Asia (*I*^2^ = 0, *p*-heterogeneity = 0.52), among participants with mean/median BMI ≥ 25 kg/m^2^ (*I*^2^ = 40.94, *p*-heterogeneity = 0.11), and studies with medium/high risk of bias (*I*^2^ = 0.02, *p*-heterogeneity = 0.52), indicating that location, participants’ weight status, and the quality of the studies could partially explain some of the observed study heterogeneity (Table 5). However, it should be noted that in all subgroups, none of the pooled estimates were statistically significant.

Four articles investigated the association between maternal fasting during Ramadan and low birth weight (birth weight <2.5 kg) [47,50,63,70]. Compared with the non-fasting group, the summary OR of low birth weight in the Ramadan fasting group was 1.37 (95% CI: 0.74, 2.53; *I*^2^ = 0.0%) (Figure 8b).

**Table 5:** Subgroup analysis of Ramadan fasting vs. birth weight relationship.

Characteristics	Number of Studies	Mean Difference, kg	95% CI, kg	*I* ^2^	*p*-For Heterogeneity
**All studies**	19	0.01	−0.06, 0.08	86.19	<0.01
**Number of participants ^a^**					
≤225	11	−0.01	−0.11, 0.10	85.07	<0.01
>225	8	0.03	−0.05, 0.12	83.13	<0.01
**Age (years old) ^a^**					
≤27.3	10	0.00	−0.11, 0.11	92.50	<0.01
>27.3	9	0.02	−0.05, 0.10	55.18	0.03
**Duration of fasting per day (hours) ^a^**					
≤15.4	11	0.01	−0.04, 0.06	56.38	0.01
>15.4	8	0.03	−0.13, 0.18	91.84	<0.01
**Region**					
Middle East/Northeast Africa	7	−0.01	−0.07, 0.06	62.38	0.02
South Asia	3	−0.01	−0.08, 0.06	0.00	0.53
Oceania/Europe	9	0.05	−0.10, 0.20	91.49	<0.01
**BMI (kg/m^2^)**					
<25	6	0.03	−0.09, 0.16	85.87	<0.01
≥25	7	0.05	−0.02, 0.11	40.94	0.11
**MMAT (risk of bias)**					
Low	12	0.02	−0.09, 0.13	90.48	<0.01
Medium/High	7	−0.01	−0.05, 0.02	0.02	0.52

^a^ Cutoffs were based on median values of the included studies. Body mass index (BMI); Mixed Method Appraisal Tool Version 2018 (MMAT).

#### 3.4.3. Other Outcomes for Ramadan Studies: Qualitative Analyses

##### Maternal Health Outcomes

We categorized other maternal health outcomes reported as hematological parameters/lipid profiles, mode of delivery, oxidative stress markers, and pregnancy complications (Table 6). Overall, findings on the associations between Ramadan fasting and these outcomes were mixed, with studies showing direct, inverse, and no associations. For example, Ramadan fasting has been associated with both higher [62] and lower risk [47] of GDM, yet other studies reported no association [64,65]. Similarly, risk of caesarean section has been reported to be both higher [62] and lower [50] for fasting mothers, yet other studies reported no association between Ramadan fasting and mode of delivery [47,48,58,59,61,64,70].

##### Fetal Outcomes

Three articles showed that Ramadan fasting in the second trimester was associated with a lower amniotic fluid index in pregnant women [51,52,70], although only one article showed statistical significance [70]. However, three other studies showed that Ramadan fasting in the third trimester was associated with a higher amniotic fluid index [59,72,73]. Most previous studies showed that Ramadan fasting was not associated with Doppler flow indices [51,52,58,59,61,77] or fetal growth [51,52,59,61,70,71,72,77] (see Table 6).

##### Birth Outcomes

Most previous studies showed that Ramadan fasting was not associated with neonatal Appearance, Pulse, Grimace, Activity, and Respiration (APGAR) score [47,55,58,62,70], risk of neonatal intensive care unit (NICU) admission [58,59,61,70], or birth anthropometry [47,57,64,65,66,67,69] (see Table 6). However, AlMogbel et al. recruited only pregnant women with GDM, and the result showed that Ramadan fasting was associated with higher risk of neonatal hyperbilirubinemia but lower risk of neonatal hypoglycemia [48].

##### Childhood Outcomes

A cohort study involved 191 children aged 4 to 13 years (Table 6). Of these, mothers of 98 children fasted throughout Ramadan during pregnancy, whereas mothers of 93 children did not fast. The results showed that, compared with the non-fasting group, Ramadan fasting during pregnancy was not associated with childhood intelligence quotient (IQ), height, or weight [49]. Since there was only one study investigating the association of maternal Ramadan fasting with childhood outcomes, a definitive conclusion could not be derived.

## 4. Discussion

### 4.1. Principal Findings

In this systematic review, the chrononutrition factors investigated in the original studies could be broadly classified into four categories: meal skipping, night eating, meal frequency, and (Ramadan) fasting. We found that meal skipping and night eating have a potential adverse influence on maternal and birth outcomes, including increased PPWR [26], GWG [30], risk of spontaneous preterm delivery [20,21], insomnia [28], impaired maternal nutritional status [22,23], and impaired glucose metabolism [25,32,33]. However, in the meal skipping literature, it is unclear whether these associations were causal, as most studies were cross-sectional in nature. Evidence on the associations of maternal meal frequency with maternal and offspring outcomes appeared mixed and inconsistent. In this meta-analysis, maternal fasting during Ramadan was associated with favorable short-term maternal outcomes (lower GWG during Ramadan and fasting blood sugar) but not birth outcomes (birth weight, gestational age, and odds of low birth weight and preterm delivery). For other associations between Ramadan fasting and maternal and offspring outcomes that could not be pooled quantitatively, our qualitative review revealed no consistent associations.

### 4.2. Meal Skipping

According to the 2007–2016 National Health and Nutrition Examination Survey, people who skipped breakfast, lunch, or dinner had a lower diet quality and a higher energy intake in subsequent meals, yet the net effect of skipped meals on total daily energy intake was still negative [79]. However, Shiraishi et al. showed that skipping breakfast lowered protein intake but did not affect total energy intake [24]. Therefore, whether nutrient intake is a confounding factor for the effects of meal skipping on maternal and birth outcomes remains to be further explored.

Two studies found that meal skipping during pregnancy can cause the pituitary gland to release corticotropin-releasing hormone (CRH), which in turn stimulates the adrenal glands to secrete and release cortisol [80]. As maternal cortisol levels increase, placental CRH production also increases, which in turn aids labor by expanding the uterus and stimulating smooth muscle contractions. However, irregular eating patterns caused by loss of appetite may be a potential sign that labor is approaching rather than meal skipping leading to labor. Therefore, the causal relationship between meal skipping and spontaneous birth requires further investigation [20,21].

### 4.3. Night Eating

In human, endogenous melatonin levels start to increase approximately 2 h before natural sleep onset and peak approximately 5 h later [81]. Since eating at night can lead to a decrease in melatonin secretion [82], it can result in poor sleep quality [28,29]. During pregnancy, the peak of maternal melatonin levels gradually increase approximately four-fold from 24 weeks of gestation to term [83]. Melatonin protects fetal growth and development through its antioxidant [84] and homeostatic effects [85] on the placenta. In animal studies, melatonin has been shown to inhibit uterine contractions by inhibiting prostaglandin synthesis [86]. Therefore, reduced melatonin secretion due to disruption of circadian rhythms by night eating behavior [82] may increase the risk of preterm birth [27].

That night-eating is associated with higher GWG and an adverse metabolic profile during pregnancy is also plausible. In studies by Gontijo et al. and Loy et al., night eating resulted in lower total energy intake but higher GWG [30] and fasting glucose levels [32] compared to day eating. In an animal study, day-eating mice (which are nocturnal in contrast to human) gained more weight and adiposity compared with night-eating mice [87]. The day-eating mice also developed hyperinsulinemia, hypercorticosteronemia, and hepatic lipid accumulation with increased lipogenic gene expression. Thus, eating at a time that is not aligned with one’s natural circadian rhythm may result in higher risk of obesity and metabolic disorders.

### 4.4. Meal Frequency

Globally, about 32.4 million pregnant women are anemic, and the rates are highest in low-income countries, especially those in Southeast Asia and Africa [88]. In our qualitative analysis, five studies from Ethiopia [41,42,43,44,45] and one study from India showed that lower meal frequency in pregnant women was associated with increased risk of anemia and preterm birth, respectively. However, since only one study showed that women with lower meal frequency had lower energy intake [36], it is unclear how lower meal frequency can cause anemia or preterm birth. It is also uncertain whether lower meal frequency is an indicator of meal skipping due to food insecurity, especially when the studies were conducted in developing countries.

A higher number of daily eating episodes was associated with higher 2 h glycemia in a cross-sectional analysis among pregnant women in a cohort study [39]. In the general population, a lower meal frequency (1–2 times compared with 3 times a day) in men was associated with higher risk of type 2 diabetes [89], but another cohort study reported no associations in women [90]. Thus, whether meal frequency is linked to blood glucose level and diabetes in the healthy population is still inconclusive. However, among patients with GDM, six meals a day led to better glycemic control than three meals a day in an isocaloric trial [40]. These data are similar to another study among type 1 diabetes patients, which observed that the number of meals was inversely associated with HbA1c and mean blood glucose measurements [91].

### 4.5. Ramadan Fasting

Sick, pregnant, and menstruating women are exempted from Ramadan fasting, yet 70–90% of pregnant women prefer to fast [16], of which 40–55% fast for the entire Ramadan period [92,93]. In our meta-analysis, compared with the non-fasting group, Ramadan fasting was associated with favorable short-term maternal outcomes (lower GWG during Ramadan, lower fasting glycemia) but not birth weight and gestational age. These health implications may be short-lived and reversed after the end of the Ramadan month, due to festive celebration after the fasting month [94]. For example, Fernando et al. showed that mean weight loss with Ramadan fasting was 1.34 kg, but most of the weight was regained a few weeks post-Ramadan [95].

While previous research and our systematic review suggest that eating at night can disrupt circadian rhythms and be harmful to offspring [27], the analysis of Ramadan fasting (which involves eating during nighttime and a prolonged fasting interval among other things) did not find similar results. The consequences of Ramadan fasting may be affected by fasting days, fasting period per day, and dietary quality and quantity during Ramadan. Ramadan fasting is a special circumstance that lasts for only one month, and its influence may thus be lesser compared to habitual dietary behavior such as non-Ramadan night eating. Of note, some studies have shown that the overall dietary quality was improved during the Ramadan month [96]. Thus, it may also be possible that a prolonged daily fasting period and improved dietary quality attenuated any potential adverse influence of night eating. However, since objective measurement of fasting is difficult, it is unclear whether fasting behavior is consistent across populations and if Muslim pregnant women have different levels of fasting exposure or other factors associated with health outcomes. Many studies investigating Ramadan fasting did not adjust for potential confounding factors, further complicating evidence consolidation.

### 4.6. Strengths, Limitations, and Future Perspectives

In this systematic review, we identified potential studies from two databases using comprehensive search terms. We also included studies investigating different dimensions of chrononutrition during pregnancy, including meal skipping, meal frequency, night eating, and (Ramadan) fasting. Thus, to our knowledge, this is by far the most comprehensive review on the study of maternal chrononutrition and its associations with both maternal and offspring outcomes. However, we are hindered by many limitations present in the original studies. For example, most of the studies were cross-sectional, meaning temporal relationships cannot be easily established due to the possibility of reverse causality. Many studies also did not provide clear definitions of chrononutrition factors, such as the number of meals skipped, fasting days, or fasting period per day, which impedes the formation of strong conclusions. Moreover, the heterogenous study designs as well as the variable and scattered exposures and outcomes investigated also hindered quantitative meta-analysis for most associations of interest.

In order to further elucidate the effect of chrononutrition factors on maternal and offspring health outcomes, larger and well-conducted cohort and interventional studies are needed. Information on covariates such as diet quality and quantity, fasting days, fasting period per day, and trimester exposure should also be collected and considered during analysis. In addition to diet, exercise [97] and sleep [98] can regulate the circadian rhythm, so the interaction between diet, sleep, exercise, and circadian rhythm can be further explored in the future. Since chrononutrition factors are interrelated, more advanced statistical techniques, for example those that consider these factors holistically as “temporal dietary patterns” [99], may help to generate further insights.

## 5. Conclusions

In this systematic review, we showed that meal skipping and night eating during pregnancy was generally related to adverse maternal and birth outcomes, whereas strong conclusions cannot be reached for meal frequency and non-Ramadan daily fasting. Ramadan fasting did not seem to affect birth weight or gestational age at birth, but evidence for other maternal and offspring outcomes was inconsistent. Most of these results are not supported by strong evidence, indicating pressing needs for further research.

## Figures and Tables

**Table 6:** Associations of Ramadan fasting with maternal and child outcomes (compared to no Ramadan fasting).

	Maternal Outcomes	Fetal Outcomes	Birth Outcomes	Childhood Outcomes
**Higher/direct association**	**Hematological parameter/lipid profile** TC [19,72] TG [19,72] Urea [19] LDL [19] Albumin [19] BUN [71] Visfatin1 [53] NGAL [71] **Mode of delivery** Risk of caesarean section [62] Risk of induction labor [62] **Oxidative stress marker** TAS [68] **Pregnant women’s health** Ketonuria [50,61] Risk of GDM [62]	**Biophysical profile** Amniotic fluid index [59,73,74] **Doppler flow indices** [71] Renal artery PI [71] Renal artery RI [71] **Placenta** Weight of placenta [57]	**Neonatal health indicator** Fifth minutes APGAR score [65] **Hematological parameter** Risk of neonatal hyperbilirubinemia * [48]	NA
**No difference/association**	**Hematological parameter/lipid profile** [65,75] Cortisol [72] TC [61] LDL [72] HDL [19,61,72] VLDL [61,72] LDL/HDL [72] RBC [75] Hb [75] Hct [75] MCV [75] MCH [75] MCHC [75] Plt [75] Risk of hypoglycemia [50] **Mode of delivery** * [47,48,58,59,61,64,70] **Oxidative stress marker** TOS [68] OSI [68] **Pregnant women’s health** Risk of GDM [64,65] Risk of pre-eclampsia [47,64] Risk of pregnancy-induced hypertension [48,62,65]	**Biophysical profile** [72,76] Amniotic fluid index [51,52,58,61,72,76] [70] (third trimester) **Doppler flow indices** [51,52,58,59,61,77] Umbilical artery S/D ratio [70,71,72] Renal artery S/D [71] **Fetal growth** Abdominal circumference [51,52,59] Fetal weight gain [51,52,59,70,72] Biparietal diameter [52,59,72] [70] (second trimester) Femur length [52,59,70,72] **Heart tracing** Heart rate [78] **Placenta** Placental location [77] Weight of placenta [64,66]	**Neonatal health indicator** First minutes APGAR score [62,65,70] Fifth minutes APGAR score [47,55,58,62,70] Risk of macrosomia * [48] Risk of admission to NICU [58,59,61,70] **Birth anthropometry** Height [47,57,64,65,66,67,69] Head circumference [47,57,64,65,66,69] Mid arm circumference [64,66]	**Brain development** IQ [49] **Children growth** Weight for age [49] Height for age [49] BMI for age [49]
**Lower/inverseassociation**	**Hematological parameter/lipid profile** TG [61] VLDL [61] Protein [19] Sirtuin1 [53] **Mode of delivery** Risk of caesarean delivery [50] **Pregnant women’s health** Risk of GDM [47]	**Biophysical profile** Amniotic fluid index [70] (second trimester) Breath movement [76] **Fetal growth** Biparietal diameter [51] Femur length [51] Head circumference [51] **Heart tracing** Large accelerations [78]	**Hematological parameter** Risk of neonatal hypoglycaemia * [48]	NA

Appearance, Pulse, Grimace, Activity, and Respiration (APGAR); blood urea nitrogen (BUN); gestational diabetes mellitus (GDM); high density lipoprotein (HDL); hemoglobin (Hb); hematocrit (Hct); intelligence quotient (IQ); low density lipoprotein (LDL); mean corpuscular hemoglobin (MCH); mean corpuscular hemoglobin concentration (MCHC); mean corpuscular volume (MCV); neutrophil gelatinase-associated lipocalin (NGAL); neonatal intensive care unit (NICU); oxidative stress index (OSI); resistance index (RI); platelet (Plt); pulsatility index (PI); red blood cell (RBC); systole/diastole (S/D); total antioxidant status (TAS); total cholesterol (TC); triglyceride (TG); total oxidant status (TOS); very low density lipoprotein (VLDL). * [48] Only recruited women with GDM.

## Data Availability

The data that support the findings of this study are available from the corresponding author upon reasonable request.

## Supplementary Materials

The following supporting information can be downloaded at: https://www.mdpi.com/article/10.3390/nu15030756/s1, Supplementary Table S1: Search strategy for the databases; Supplementary Table S2: Risk of bias assessment_MMAT; Supplementary Table S3: Characteristics of studies on Ramadan fasting in relation to maternal and birth outcomes.
