# Supplementary material for: Chrononutrition during Pregnancy and Its Association with Maternal and Offspring Outcomes: A Systematic Review and Meta-Analysis of Ramadan and Non-Ramadan Studies
Source: Nutrients. 2023 Feb 2;15(3):756. doi: 10.3390/nu15030756 (PMC9921927; doi:10.3390/nu15030756)
Supplement: Supplementary file 1 [file nutrients-15-00756-s001.zip › nutrients-2105863-supplementary.pdf]

Supplementary Table S1. Search strategy for the databases

Supplementary Table S2. Risk of bias assessment_MMAT

Supplementary Table S3. Characteristics of studies on Ramadan fasting in relation to maternal and birth outcomes.

Supplementary Table S1. Search strategy for the databases

Databases	Populations	Interventions
PubMed	(Pregnancy [tw] OR Pregnant [tw] OR Gravidity [tw] OR Gestation [tw] OR Antenatal [tw] OR Preconception OR Pregnancy [mh] OR Postpartum [tw] OR Postnatal [tw] OR Mother [tw] OR Maternal OR Mother [mh])	(Chrononutrition [tw] OR Intermittent fasting [tw] OR Maternal fasting [tw] OR Meal timing [tw] OR Meal frequency [tw] OR Meal skipping [tw] OR Eating time [tw] OR Breakfast skipping [tw] OR Lunch skipping [tw] OR Dinner skipping [tw] OR Night-time eating [tw] OR Time-restricted eating [tw] OR Diet timetable [tw] OR Food timetable [tw] OR Time-restricted feeding [tw] OR Ramadan fasting [tw] OR Muslim fasting [tw] OR Regular eating [tw] OR Eating rate [tw] OR Temporal eating [tw] OR Diurnal eating [tw] OR Nocturnal eating [tw] OR Night eating [tw] OR Delayed meal [tw] OR Circadian eating [tw] OR Meal pattern [tw] OR Eating episode [tw])
Embase	('pregnancy'/exp OR pregnancy OR 'child bearing' OR 'childbearing' OR 'gestation' OR 'gravidity' OR 'intrauterine pregnancy' OR 'labor presentation' OR 'labour presentation' OR 'pregnancy' OR 'pregnancy maintenance' OR 'pregnancy trimesters' OR pregnant OR antenatal OR preconception OR postpartum OR postnatal OR 'mother'/exp OR mother OR 'mother' OR 'motherhood' OR 'mothering' OR 'mothers' OR 'maternal'/exp OR maternal)	('chrononutrition'/exp OR chrononutrition OR 'intermittent fasting'/exp OR 'intermittent fasting' OR 'maternal fasting' OR 'meal timing' OR 'meal frequency'/exp OR 'meal frequency' OR 'meal skipping'/exp OR 'meal skipping' OR 'skipping a meal' OR 'skipping meals' OR 'eating time' OR 'breakfast skipping'/exp OR 'breakfast skipping' OR 'skipping breakfast' OR 'lunch skipping' OR 'dinner skipping' OR 'night-time eating' OR 'time restricted eating'/exp OR 'time restricted eating' OR 'diet timetable' OR 'food timetable' OR 'time restricted feeding'/exp OR 'time restricted feeding' OR 'ramadan fasting'/exp OR 'islamic fast' OR 'islamic fasting' OR 'muslim fasting' OR 'ramadan fast' OR 'ramadan fasting' OR 'regular eating' OR 'eating rate' OR 'temporal eating' OR 'diurnal eating' OR 'nocturnal eating' OR 'night eating' OR 'delayed meal' OR 'circadian eating' OR 'meal pattern' OR 'eating episode')

Supplementary Table S2. Risk of bias assessment_MMAT

Quantitative non-randomized studies

Authors, publication date	Are the participants representative of the target population?	Are measurements appropriate regarding both the outcome and intervention (or exposure)?	Are there complete outcome data?	Are the confounders accounted for in the design and analysis?	During the study period, is the intervention administered (or exposure occurred) as intended?
Parveen et al., 2020	Yes	Yes	Yes	No	Yes
Savitri et al., 2018	Yes	Yes	Yes	Yes	Yes
Safari et al., 2019	No	No	No	Yes	No
Engin-Ustun et al., 2016	Yes	Yes	Yes	Yes	Yes
AlMogbel et al., 2022	Yes	Yes	Yes	Yes	Yes
Hossain et al., 2021	Yes	Yes	Yes	No	Yes
Gul et al., 2018	Yes	Yes	No	No	Yes
Sakar et al., 2016	Yes	Yes	Yes	No	No
Baynouna et al., 2014	Yes	Yes	No	No	Yes
Sakar et al., 2015	Yes	Yes	Yes	No	Yes
Hassanein et al., 2021	Yes	Yes	Yes	No	Yes
Moradi., 2011	Yes	Yes	Yes	No	Yes
Abd-Allah Rezk et al., 2016	Yes	No	Yes	No	Yes
Makvandi et al., 2019	Yes	Yes	Yes	No	Yes
Seckin et al., 2014	Yes	Yes	Yes	No	Yes
Savitri et al., 2014	Yes	Yes	Yes	Yes	Yes
Awwad et al., 2012	Yes	Yes	Yes	Yes	Yes
Gur et al., 2015	Yes	Yes	Yes	Yes	Yes
Hizli et al., 2012	Yes	No	Yes	No	Yes
Khoshdel et al., 2014	Yes	Yes	Yes	Yes	Yes
Ozturk et al., 2011	Yes	Yes	Yes	Yes	Yes
Ziaee et al., 2010	Yes	No	No	No	Yes

Khoshdel et al., 2014	Yes	No	No	No	Yes
Petherick et al., 2014	Yes	Yes	Yes	Yes	Yes
Kavehmanesh et al., 2004	Yes	Yes	Yes	No	Yes
Karateke et al., 2015	Yes	Yes	Yes	Yes	Yes
Bayoglu Tekin et al., 2016	Yes	Yes	Yes	Yes	No
Mirghani et al., 2004	Yes	Yes	Yes	No	No
Afandi et al., 2017	Yes	Yes	Yes	No	No
Mirghani., 2005	Yes	Yes	No	No	No
Malhotra et al., 1989	Yes	Yes	Yes	Yes	Yes
Mirghani et al., 2007	Yes	Yes	Yes	No	No
Kiziltan et al., 2005	Yes	Yes	Yes	Yes	Yes
Khalaf et al., 2015	Yes	No	No	No	No
Azizi et al., 2004	Yes	Yes	Yes	Yes	Yes
Kamyabi et al., 2004	Yes	Yes	Yes	No	Yes
Ismail et al., 2011	Yes	Yes	Yes	Yes	Yes
Dikensoy et al., 2009	Yes	No	Yes	No	No
Rakicioğlu et al., 2006	Yes	No	Yes	Yes	Yes
Mirghani et al., 2006	Yes	No	Yes	No	No
Mirghani et al., 2003	Yes	Yes	Yes	No	No
Hernández-Díaz et al.,2014	Yes	Yes	Yes	Yes	Yes
Nulty et al., 2021	Yes	No	Yes	Yes	Yes
Shiraishi et al., 2019	Yes	No	Yes	No	Yes
Shemsu et al., 2020	Yes	No	Yes	No	Yes
Celik et al., 2018	Yes	Yes	Yes	Yes	No
Fite et al., 2022	No	Yes	Yes	No	Yes
Loy et al.,2019	Yes	Yes	Yes	Yes	Yes
Loy et al., 2017	Yes	Yes	Yes	Yes	Yes

Dong et al., 2020	Yes	Yes	Yes	Yes	Yes
Salunkhe et al.,2018	Yes	Yes	Yes	No	Yes
Kedir et al., 2021	Yes	Yes	Yes	Yes	Yes
Debella et al., 2021	Yes	Yes	Yes	Yes	Yes
Grum et al., 2018	Yes	Yes	Yes	Yes	Yes
Abriha et al., 2014	Yes	Yes	Yes	Yes	Yes
Gebre et al., 2015	Yes	Yes	No	Yes	Yes
Englund-Ögge et al., 2017	Yes	Yes	Yes	Yes	Yes
Ainscough et al., 2020	Yes	Yes	Yes	Yes	Yes
Loy et al., 2020	Yes	Yes	Yes	Yes	Yes
Loy et al., 2016	Yes	Yes	Yes	Yes	Yes
Gontijo et al., 2020	Yes	Yes	Yes	Yes	Yes
Deniz et al., 2019	Yes	Yes	Yes	No	Yes
Ku et al., 2022	Yes	Yes	Yes	Yes	Yes
Wolynczyk-Gmaj et al.,2017	Yes	Yes	Yes	Yes	Yes
Tug et al., 2011	Yes	Yes	Yes	Yes	Yes
Abd-El-Aal et al., 2019	Yes	No	Yes	Yes	Yes

Randomized controlled clinical trial

**Authors, publication date**	**Is randomization appropriately performed?**	**Are the groups comparable at baseline?**	**Are there complete outcome data?**	**Are outcome assessors blinded to the intervention provided?**	**Did the participants adhere to the assigned intervention?**
**Yong et al., 2022**	**No**	**Yes**	**Yes**	**No**	**Yes**
**Messika et al., 2022**	**Yes**	**Yes**	**Yes**	**Yes**	**Yes**

Supplementary Table S3. Characteristics of studies on Ramadan fasting in relation to maternal and birth outcomes.

Authors, publication date	Study design	Population	Number of Participants	The period of Ramadan	Duration of fasting per day (for Ramadan)	Participants' Age	BMI	Comparison of exposure	Outcome	Period of exposure assessment	Covariates	Main findings
Parveen (Pakistan, 2020) [1]	Cross-sectional study	Healthy pregnant women	226<br>1. Fasting >15 days: 58<br>2. Fasting <15 days: 168	5/5/2019-6/4/2019	15 hours	1. Fasting >15 days: 28.4<br>2. Fasting <15 days:28.1 (Mean)	1. Fasting >15 days: 25.7<br>2. Fasting <15 days: 25.9 (During pregnancy) (Mean)	Fasting >15 days vs. Fasting <15 days	Mode of delivery, GDM, pre-eclampsia, preterm birth, birth weight, birth height, head circumference, mid arm circumference, weight of placenta	15-40 weeks of gestation	na	Fasting women were not found to have poor maternal and fetal outcomes when compared to not fasting women.
Savitri (Indonesia, 2018) [2]	Cohort study	Pregnant women	139<br>1. Fasting ≥1 days: 110<br>2. Fasting = 0 days: 29	7/21/2012-8/18/2012<br>7/10/2013-8/7/2013<br>6/29/2014-7/27/2014	14.3 hours	1. Fasting ≥1 days: 28.8<br>2. Fasting = 0 days: 27.4 (Mean)	1. Fasting ≥1 days: 22.6<br>2. Fasting = 0 days: 21.1 (Pre-pregnancy) (Median)	Fasting during the first & second &third trimester vs. Not fasting	Birth weight	1-40 ↑ weeks of gestation (The first/second/third trimester)	Secondhand smoking exposure, family income categories, maternal education categories, pre-pregnancy BMI,	Maternal fasting did not seem to affect the birth weight of newborns.

											gestational duration, parity.	
Safari (Iraqi kurdistan, 2019) [3]	Case-control study	Healthy pregnant women	299<br>1. Ramadan fasting: 155<br>2.No Ramadan fasting: 144	5/27/2017-6/25/2017	18 hours	1. Ramadan fasting: 27.87<br>2. No Ramadan fasting: 27.08<br>(Mean)	1. Ramadan fasting<br>2.6% Underweight<br>55.3% Normal<br>32.9% Overweight<br>9.2% Obese<br>2. No Ramadan fasting<br>6.3% Underweight<br>58% Normal<br>29.4% Overweight<br>6.3% Obese<br>(Pre-pregnancy)	Fasting vs. Not fasting	Mode of delivery, GDM, pre-eclampsia, preterm birth, low birth weight, birth height, head circumference, 5th minutes APGAR score	17-29 weeks of gestation (The second trimester)	Age, maternal education, maternal occupation, number of paras, BMI before pregnancy	Fasting during the second trimester of the pregnancy decreased the risk of GDM and excessive weight gain during pregnancy.
Engin-Ustun (Turkey, 2016) [4]	Case-control study	Healthy pregnant women	36<br>1. Fasting ≥10 days: 18	na	Na	1. Fasting ≥10 days: 29.2<br>2. No Ramadan	1. Fasting ≥10 days: 27.7<br>2. No Ramadan	Number of fasting days vs. sirtuin	serum sirtuin, visfatin levels, hematological parameter	13-40↑ weeks of gestation (The second	BMI, pregnancy weeks, ages	A correlation analysis provided a negative correlation between number of fasting days and sirtuin

			2. No Ramadan fasting: 18			fasting: 26.8 (Mean)	fasting: 27 (Mean) (During pregnancy)	& visfatin levels		& third trimester)		levels and a positive correlation with visfatin levels.
AlMogbel (Australia, 2022) [5]	Cohort study	Pregnant women with GDM	293<br>1. Not fasting: 48<br>2. Fasting 12-13 h/d: 31<br>3. Fasting 13-14 h/d: 85<br>3. Fasting 14-15 h/d: 43<br>4. Fasting 15-16 h/d: 86	na	12-16 hours	1. Not fasting: 30.8<br>2. Fasting 12-13 h/d: 31.6<br>3. Fasting 13-14 h/d: 31.6<br>4. Fasting 14-15 h/d: 29.9<br>5. Fasting 15-16 h/d: 31.4 (Mean)	1. Not fasting: 25.2<br>2. Fasting 12-13 h/d: 27.0<br>3. Fasting 13-14 h/d: 26.1<br>4. Fasting 14-15 h/d: 26.7<br>5. Fasting 15-16 h/d: 26.6 (Mean) (pregnancy)	1. Not fasting<br>2. Fasting 12-13 h/d<br>3. Fasting 13-14 h/d<br>4. Fasting 14-15 h/d<br>5. Fasting 15-16 h/d	Gestational HTN, weight gain during pregnancy birth weight, hypoglycaemia, hyperbilirubinemia, preterm birth, mode of delivery	1-40 ↑ weeks of gestation (The first& second & third trimester)	Gestational age at delivery, gender, ethnicity and birthweight centile, gestational age at diagnosis, insulin treatment and birthweight centile, insulin treatment and LGA	No significant effect of Ramadan exposure on mean birthweight, macrosomia and maternal outcomes. But neonatal hypoglycaemia decreased for the fasting period of more than 15 h/d group. and neonatal hyperbilirubinemia increased for the group of fasting more than 21 days.
Hossain (India, 2021) [6]	Case-control study	Healthy pregnant women	215<br>1. Fasting >7 days: 123<br>2. No Ramadan fasting: 92	4/25/2020-	14-15 hours	1. Fasting >7 days: 26.54<br>2. No Ramadan fasting: 26.84 (Mean)	1. Fasting >7 days: 25.44<br>2. No Ramadan fasting: 23.97 (Mean) (At start of pregnancy)	Fasting >7 days vs. Not fasting	Birth weight, birth height, head circumference, 1&55th minutes APGAR score	(Definition unclear)	na	Ramadan fasting did not affect maternal outcomes during pregnancy.

Gul (Pakistan, 2018) [7]	Case-control study	Healthy pregnant women	180<br>1. Fasting >10 days: 100<br>2. No Ramadan fasting: 80	5/28/2017-6/28/2017	15 hours	1. Fasting >10 days: 27.16<br>2. No Ramadan fasting: 27.36<br>(Mean)	1. Fasting >10 days: 25.31<br>2. No Ramadan fasting: 25.64<br>(Mean)<br>(Definition unclear)	Fasting >10 days vs. Not fasting	Preterm birth, birth weight, birth height, head circumference, mid arm circumference, weight of placenta	1-40↑ weeks of gestation (Definition unclear)	na	Ramadan fasting did not affect babies' outcomes during pregnancy.
Sakar (Turkey, 2016) [8]	Case-control study	Healthy pregnant women	338<br>1. Ramadan fasting : 168<br>2. No Ramadan fasting: 170	2013	17.7 hours	1. Ramadan fasting: 28.04<br>2. No Ramadan fasting: 27.55<br>(Mean)	1. Ramadan fasting: 27.91<br>2. No Ramadan fasting:27.47<br>(Mean)<br>(Definition unclear)	Fasting vs. Not fasting	Birth weight, birth height, head circumference, weight of placenta	25 ↑ weeks of gestation (The third trimester)	na	The mean placental weight in the fasting group was significantly higher. In addition, a significant correlation between placental weight and maternal serum albumin level was observed in the fasting group.
Baynouna (United Arab Emirates, 2014) [9]	Cross-sectional study	Healthy pregnant women	150<br>1. Fasting 30 days: 76<br>2. No Ramadan fasting: 74	2010	14 hours	1. Fasting 30 days: 28.4<br>2. No Ramadan fasting: 27.5<br>(Mean)	1. Fasting 30 days: 28.7<br>2. No Ramadan fasting: 28.6<br>(Mean)<br>(Definition unclear)	Fasting 30 days vs. Not fasting	Fasting blood sugar, postprandial blood sugar	20-36 weeks of gestation	Age, parity, and gestational age	The mean random blood glucose level after 1 hour of breaking the fast was significantly higher in the fasting group than not fasting group

Sakar (Turkey, 2015) [10]	Case-control study	Healthy pregnant women	106<br>1. Fasting ≥15 days: 52<br>2. No Ramadan fasting: 54	7/9/2013-8/7/2013	18.3 hours	1. Fasting ≥15 days: 26.8<br>2. No Ramadan fasting: 28.4<br>(Mean)	na	Fasting ≥15 days<br>vs.<br>Not fasting	Amniotic fluid index, Doppler flow indices, fetal abdominal circumference, fetal weight gain, weight gain during Ramadan	≥13 weeks of gestation (The second & third trimester)	na	At the end of the Ramadan, increase in biparietal diameter, head circumference, and femur length showed a statistically significant difference from initial measurements. When fasting and not fasting groups were compared separately, an increase in amniotic fluid index was statistically significant in the non-fasting group.
Hassanein (United Arab Emirates, 2021) [11]	Cohort study	Pregnant women with GDM	25<br>1. Fasting 1-10 days: 7<br>2. Fasting 11-20 days: 1<br>3. Fasting 21-30 days: 17	2016	na	31.8<br>(Mean)	na	Pre Ramadan vs. Post Ramadan	Blood glucose	≥24 weeks of gestation	na	The average glucose improved significantly, while time in target and percent above target numerically improved during Ramadan compared to pre-Ramadan. There was significant increment on the number of hypoglycemic events in Ramadan.
Moradi (Iran, 2011) [12]	Case-control study	Healthy pregnant women	52<br>1. Fasting ≥15 days: 25	8/21/2009-9/23/2009	15.3 hours	1. Fasting ≥15 days: 28.3<br>2. No Ramadan	na	Fasting ≥15 days	Amniotic fluid index, Doppler flow indices,	≥13 weeks of gestation (The second	na	The results showed that Ramadan fasting has no adverse effect on fetal

			2. No Ramadan fasting: 27			fasting: 28.3 (Mean)		vs. Not fasting	fetal abdominal circumference, fetal weight fetal length, gain, weight gain during Ramadan	& third trimester)		growth, amniotic fluid volume or maternofetal circulation.
Abd-Allah Rezk (Egypt, 2016) [13]	Case-control study	Healthy pregnant women	450<br>1. Fasting 30 days: 210<br>2. No Ramadan fasting: 240	6/17/2015-7/16/2015	12-16 hours Average: 14.6 hours)	1. Fasting 30 days: 23.2<br>2. No Ramadan fasting: 23.0 (Mean)	1. Fasting 30 days: 25.2<br>2. No Ramadan fasting: 24.8 (Mean) (Definition unclear)	Fasting 30 days vs. Not fasting	Birth weight, mode of delivery, amniotic fluid index, Doppler flow indices, 5th minutes APGAR score, risk of admission to NICU	36-40 weeks of gestation (The third trimester)	na	Short-term maternal fasting had no deleterious effect on fetal well-being parameters or neonatal outcome.
Makvandi (Iran, 2019) [14]	Case-control study	Healthy pregnant women	200<br>1. Ramadan fasting (average 12.6 days): 100<br>2. No Ramadan fasting (Definition unclear): 100	2014	15.8 hours	1. Ramadan fasting: 26.23<br>2. No Ramadan fasting: 26.27 (Mean)	na	Ramadan fasting (average 12.6 days) vs. Not fasting	Hematological parameter, gestational age at birth, mode of delivery	≥13 weeks of gestation (The second & third trimester)	na	This study concluded that partial fasting during Ramadan from a hematological point of view was of no concern for pregnant women 3 months after the end of Ramadan.

Seckin (Turkey, 2014) [15]	Case-control study	Healthy pregnant women	169<br>1. Fasting ≥20 days: 82<br>2. No Ramadan fasting: 87	na	18.7 hours (average)	1. Fasting ≥20 days: 24<br>2. No Ramadan fasting: 26.1 (Mean)	na	Fasting ≥20 days vs. Not fasting	Amniotic fluid index, Doppler flow indices, fetal abdominal circumference, fetal weight fetal length, gain, birth weight, mode of delivery, risk of admission to NICU	≥29 weeks of gestation	Maternal age, parity, and gestational age.	Fetal development, Doppler parameters, and neonatal outcomes were not significantly affected in healthy fasting women; however, there was a significant association between fasting and amniotic fluid index.
Savitri (Netherlands, 2014) [16]	Cohort study	Pregnant women	130<br>1. Fasting ≤15 days: 21<br>2. Fasting >15 days: 49<br>3. No Ramadan fasting: 60	8/11/2010-9/10/2010	14 hours	1. Fasting ≤15 days: 28.9<br>2. Fasting >15 days: 28.9<br>3. No Ramadan fasting: 29.3 (Mean)	na	1. Fasting ≤15 days & Fasting >15 days vs. Not fasting<br>2. Fasted in first & second & third trimesters vs. Not fasting	Birth weight	≥4 weeks of gestation	Maternal smoking status during Ramadan and pregnancy, maternal ethnicity, nulliparity, risk classification at the start of prenatal care and for socio-economic factors	There were no differences in birth weight between newborn whose mothers had or had not fasted if Ramadan fasting had taken place later in pregnancy. Ramadan fasting during early pregnancy may lead to lower birth weight of newborns.

Awwad (Lebanon, 2012) [17]	Cohort study	Healthy pregnant women.	402<br>1. Ramadan fasting: 201<br>2. No Ramadan fasting: 201	9/1/2008-9/30/2008	15.1 hours	1. Ramadan fasting: 29.7<br>2. No Ramadan fasting: 30.0<br>(Mean)	1. Ramadan fasting: 24.8<br>2. No Ramadan fasting: 24.3<br>(Mean)<br>(Definition unclear)	Fasting days<br>vs.<br>Not fasting	Preterm birth, gestational age at birth, birth weight, low birth weight, ketonuria	20-34 weeks of gestation	Maternal age, gestational age, parity, BMI	Fasting during the month of Ramadan did not seem to increase the baseline risk of preterm delivery in pregnant women regardless of the gestational age during which this practice was observed.
Gur (Turkey, 2015) [18]	Cohort study	Healthy pregnant women	156<br>1. Fasting ≥15 days: 78<br>2. No Ramadan fasting: 78	2012, 2013	16 hours	1. Fasting ≥15 days: 24.2<br>2. No Ramadan fasting: 25.1<br>(Mean)	1. Fasting ≥15 days: 24.5<br>2. No Ramadan fasting: 24.8<br>(Mean)<br>(Definition unclear)	1. Fasting ≥15 days<br>vs.<br>Not fasting<br>2. Finish day of fasting vs. Fifteenth day of fasting	Gestational age at birth, 5th minutes APGAR score, hematological parameter, amniotic fluid index, biparietal diameter, fetal abdominal circumference, head circumference fetal length	12-28 weeks of gestation	Age, parity, and gestational age	High-density lipoprotein, ferritin significantly increased, and glycated hemoglobin, insulin, and homeostasis model index significantly decreased.
Hizli (Turkey, 2012) [19]	Case-control study	Healthy pregnant women	110<br>1. Fasting ≥10 days: 56	8/11/2010-9/9/2010	15.3 hours	1. Fasting ≥10 days: 27<br>2. No Ramadan	1. Fasting ≥10 days: 25<br>2. No Ramadan	Fasting ≥10 days<br>vs.<br>Not fasting	Hematological parameter, ketonuria, mode of delivery,	≥28 weeks of gestation	Age, parity, and gestational age	Fasting of healthy women during pregnancy seemed to have no adverse effects on amniotic fluid index, fetal

			2. No Ramadan fasting: 54			fasting: 25.8 (Mean)	fasting: 27 (Definition unclear) (Definition unclear)		amniotic fluid index, Doppler flow indices, risk of admission to NICU, birth weight, gestational age at birth			Doppler and delivery parameters.
Khoshdel (Iran, 2014) [20]	Cross-sectional study	Healthy pregnant women	39	2012/7/21-2012/8/18	16.5 hours	26.9 (Mean)	25 (Mean) (at the beginning of the study)	The data of fasting first week, second week, fourth week, two weeks after Ramadan were compared with each other	Leptin, neuropeptide Y, insulin level.	7-35 weeks of gestation	na	The result of this study revealed the important role of leptin and neuropeptide Y in the long-term regulation of energy balance in pregnant women with chronic diurnal fasting, and it further revealed that Ramadan fasting did not significantly change the serum insulin level.
Ozturk (Turkey, 2011) [21]	Case-control study	Healthy pregnant women	72<br>1. Ramadan fasting: 42	1–29 September 2008	12 hours	1. Ramadan fasting: 30.1<br>2. No Ramadan	na	Fasting vs. Not fasting	Maternal weight gain, birth	13-24 weeks of gestation	Age, parity and gestational age.	Maternal fasting during Ramadan in the second trimester did not have a

			2. No Ramadan fasting: 30			fasting: 29.5 (Mean)			weight, TAS, TOS, OSI	(The second trimester)		significant effect on maternal oxidative stress, fetal development or fetal birth weight.
Ziaee (Iran, 2010) [22]	Cohort study	Healthy pregnant women	189<br>1. Fasting 1-10 days: 28<br>2. Fasting 11-20 days: 35<br>3. Fasting >20 days: 60<br>&<br>1. First Trimester: 82<br>2. Second Trimester: 56<br>3. Third Trimester: 51<br>&<br>1. No Ramadan fasting: 66	10/15/2004-11/13/2004	13.6 hours	25.9 (Mean)	23.9 (Mean)	1. Compare fasting 0, 1-10, 11-20, >20 days with each other<br>&<br>2. Compare first/ second/third trimester with each other	Birth weight, birth length, head circumference,	1-40 ↑ weeks of gestation (The first/second/third trimester)	na	In healthy women with appropriate nutrition, Islamic fasting has no inappropriate effect on intrauterine growth and birth-time indices. Meanwhile, relative risk of low weight birth was 1.5 times in mothers on fasting at first trimester as compared to non-fasting mothers.
Khoshdel (Iran, 2014) [20]	Self-control study cohort	Pregnant women	30	7/20/2012-8/18/2012	17.1 hours	26.9 (Mean)	25 (Mean) (at the beginning of the study)	The data of fasting first week, second week, fourth	LH, FSH, oestrogen, progesterone and leptin	(Definition unclear)	na	They found poor weight gain and hypoleptinaemia in pregnant fasted women during the study.

								week, two weeks after Ramadan were compared with each other				
Petherick (England, 2014) [23]	Cohort study	Pregnant women	300<br>1. Ramadan fasting: 128<br>2. No Ramadan fasting: 172	8/11/2010-9/8/2010	18 hours	1. Ramadan fasting: 27.6<br>2. No Ramadan fasting: 29.0 (Mean)	1. Ramadan fasting 7.8% Underweight 28.9% Normal 24.2% Overweight 29.7% Obese 9.4% Missing<br>2. No Ramadan fasting 5.8% Underweight 40.1% Normal 26.7% Overweight	Fasting vs. Not fasting	Preterm birth, low birth weight, birth weight	1-40 ↑ weeks of gestation (The first/second/third trimester)	Age, fasting, trimester of exposure to fasting, maternal education, parity, booking BMI, height, baby's gender and smoking	Fasting was not found to be associated with adverse birth outcomes in this study.

							16.9% Obese 10.5% Missing (at booking)					
Kavehmanesh (Iran, 2004) [24]	Cohort study	Healthy pregnant women	539 1. Fasting ≥10 days: 284 2. Fasting <10 days: 255	na	13 hours	1. Fasting ≥10 days: 28 2. Fasting <10 days: 27 (Mean)	1. Fasting ≥10 days: 25.9 2. Fasting <10 days: 24.2 (Mean)	Fasting ≥10 days vs. Fasting <10 days	Birth weight, birth height	(Definition unclear)	na	Maternal fasting during Ramadan did not have a significant effect on the neonatal birth weight
Karateke (Turkey, 2015) [25]	Case-control study	Healthy pregnant women	1. First Trimester fasting: 40 2. Second Trimester fasting: 40 3. Third Trimester fasting: 40 & 1. First Trimester no fasting: 40 2. Second Trimester no fasting: 40 3. Third Trimester no fasting: 40	6/28/2014-7/27/2014	18.1 hours	1. First Trimester fasting: 24.7 2. Second Trimester fasting: 26.5 3. Third Trimester fasting: 27.5 & 1. First Trimester no fasting: 25.3 2. Second Trimester no fasting: 27.3 3. Third	1. First Trimester fasting: 21.2 2. Second Trimester fasting: 23.1 3. Third Trimester fasting: 22.5 & 1. First Trimester no fasting: 20.8 2. Second Trimester no fasting: 22.9 3. Third	1. First Trimester fasting vs. Not fasting 2. Second Trimester fasting vs. Not fasting 3. Third Trimester fasting vs. Not fasting	Mode of delivery, amniotic fluid index, Doppler flow indices, fetal weight gain, biparietal diameter, femur length, 1 & 5 th minutes APGAR score, risk of admission to NICU, birth weight, low birth weight,	1-40 ↑ weeks of gestation (The first/second/third trimester)	gestational age at Ramadan	There was no bad fetal outcome between pregnant women with fasting and pregnant women without fasting.

						Trimester no fasting: 29.2 (Mean)	Trimester no fasting: 21.5		maternal weight gain			
Bayoglu Tekin (Turkey, 2016) [26]	Cross-sectional study	Healthy pregnant women	48<br>1. Ramadan fasting: 23<br>2. No Ramadan fasting: 25	2014	17.24 hours	1. Ramadan fasting: 27.8<br>2. No Ramadan fasting: 30.7 (Mean)	1. Ramadan fasting: 27.3<br>2. No Ramadan fasting: 28.1 (Mean) (Definition unclear)	Fasting vs. Not fasting	Hematological parameter, Doppler flow indices, mode of delivery, birth weight	>30 weeks of gestation	Age, gestation and parity	Blood urea nitrogen, potassium and hematocrit levels, blood and urine NGAL levels were significantly higher, and fRA Doppler indices increased in fasting women during the second visit in the last week of the Ramadan, while non-fasting women had no significant alterations in each evaluation
Mirghani (United Arab Emirates, 2004) [27]	Cross-sectional study	Healthy pregnant women	63	na	na	27.4 (Mean)	na	Fasting vs. Post-meal	Fetal breathing movement	>30 weeks of gestation	na	Intermittent maternal fasting was associated with a significant alteration in the frequency and pattern of human fetal breathing movement.
Afandi (United Arab Emirates, 2017) [28]	Cohort study	Pregnant women with GDM	32<br>1. Pre-Ramadan diet (on diet): 10<br>2. Ramadan diet: 13	5/27/2017-6/24/2017	16.5 hours	32.9 (Mean)	29.5 (Mean) (pre-pregnancy)	Compare pre-Ramadan diet (on diet),	Blood glucose	(Definition unclear)	na	Ramadan fasting in women with GDM treated with diet alone or with diet plus metformin was associated with lower mean glucose

			3. Ramadan diet and metformin: 9					Ramadan diet, Ramadan diet with metformin with each other				levels and higher rates of hypoglycemia when compared with non-fasting glucose levels.
Mirghani (United Arab Emirates, 2005) [29]	Cross-sectional study	Healthy pregnant women	153<br>1. Ramadan fasting: 78<br>2. No Ramadan fasting: 75	10/29/2003-11/23/2003	13.9 hours	na	na	Fasting vs. Not fasting	Fetal heart rate	>30 weeks of gestation	Age, parity and gestational age.	The number of large accelerations in computerized fetal heart tracing was decreased in pregnant women abstaining from food and water.
Malhotra (England, 1989) [30]	Case-control study	Pregnant women	22<br>1. Ramadan fasting: 11<br>2. No Ramadan fasting: 11	4/29/1987-5/28/1987	17 hours	1. Ramadan fasting: 26.7<br>2. No Ramadan fasting: 26.6 (Mean)	na	Fasting vs. Not fasting	Hematological parameter, birth weight, 1&5th minutes APGAR score, head circumference, weight of placenta	28 weeks of gestation	Gestation age	At the end of the Ramadan fast day there was a significant fall in glucose, insulin, lactate and carnitine, and a rise in triglyceride, non-esterified fatty acid and 3-hydroxybutyrate. When compared with the control group, none of the Ramadan mothers had a completely normal set of biochemical values at the end of the fast day.

Mirghani (United Arab Emirates, 2007) [31]	Cross-sectional study	Healthy pregnant women	106<br>1. Ramadan fasting: 53<br>2. No Ramadan fasting: 53	10/4/2005-11/2/2005	14.3 hours	1. Ramadan fasting: 28.3<br>2. No Ramadan fasting: 26.4<br>(Mean)	na	Fasting vs. Not fasting	Doppler flow induces, blood glucose	(Definition unclear)	Age, parity and gestational age	Maternal fasting was not associated with significant changes in the uterine artery Doppler flow velocimetry.
Kiziltan (Turkey, 2005) [32]	Cross-sectional study	Healthy pregnant women	98<br>1. First Trimester fasting: 12<br>2. Second Trimester fasting: 29<br>3. Third Trimester fasting: 8<br>&<br>1. First Trimester no fasting: 12<br>2. Second Trimester no fasting: 30<br>3. Third Trimester no fasting: 7	10/15/2004-11/13/2004	12 hours	1. First Trimester fasting (1 month): 25.2<br>2. Second Trimester fasting (1 month): 25.1<br>3. Third Trimester fasting (1 month): 22.6<br>&<br>1. First Trimester no fasting: 26.1<br>2. Second Trimester no fasting: 24.9<br>3. Third Trimester no	1. First Trimester fasting (1 month): 25.9<br>2. Second Trimester fasting (1 month): 26.5<br>3. Third Trimester fasting (1 month): 29.1<br>&<br>1. First Trimester no fasting: 26.3<br>2. Second Trimester no fasting: 27.7<br>3. Third Trimester no	1. First Trimester fasting vs. Not fasting<br>2. Second Trimester fasting vs. Not fasting<br>3. Third Trimester fasting vs. Not fasting	Hematological parameter, weight gain during Ramadan	1-40 ↑ weeks of gestation (The first/second/third trimester)	na	Ramadan fasting had no significant adverse effect on the health of pregnant women.

						fasting: 23.8 (Mean)	fasting: 29.2 (Mean) (During gnancy)					
Khalaf (Egypt, 2015) [33]	Cross-sectional study	Healthy pregnant women	221<br>1. Ramadan fasting: 97<br>2. No Ramadan fasting: 124	7/21/2012-8/17/2012 & 7/12/2013-8/6/2013	16.4 hours (10-12 hours)	29.3 (The number of people is error)	na	Fasting vs. Not fasting	Amniotic fluid index	>36 weeks of gestation	na	The fasting in Ramadan has no effect on or may improve the amniotic fluid index in the last month, while gravidity plays a role in the amniotic fluid index in fasting group.
Azizi (Iran, 2004) [34]	Cohort study	4-13y children and whose mothers fasted at least 27 days or not fasted in Ramadan during their pregnancies	190<br>1. Fasting ≥27 days: 95<br>2. No Ramadan fasting: 95	1988-1997	13-16 hours	Children: 8.6<br>Mothers: 26.5 (pregnant) (Mean)	na	1. Fasting ≥27 days<br>2. No Ramadan fasting	Weight, height, BMI for age, IQ	≥25 weeks of gestation (The third trimester)	Father's educational level, residential status,breast-feeding duration, and percentage of Caesarean sect	Fasting during gestation did not adversely affect IQ of children whose mothers had fasted during Ramadan while being pregnant.
Kamyabi (Iran, 2004) [35]	Cross-sectional study	Healthy pregnant women	53<br>1. Fasting ≥20 days: 25<br>2. No Ramadan fasting: 28	na	12 hours	1. Fasting ≥20 days: 25.8<br>2. No Ramadan fasting: 24.4 (Mean)	na	1. Fasting ≥20 days<br>2. No Ramadan fasting	Amniotic fluid index, blood pressure	20-36 weeks of gestation	na	Fasting in Ramadan had no significant effect on the decrease of amniotic fluid index, deepest vertical pocket and amniotic fluid volume.

Ismail (Malaysia, 2011) [36]	Cohort study	Pregnant women with GDM or T2DM	37<br>1. T2DM: 24<br>2. GDM: 13	9/14/2007-10/13/2007<br>9/2/2008-10/1/2008<br>8/23/2009-9/20/2009	14.4 hours	1. T2DM: 32.3<br>2. GDM: 31.8<br>(Mean)	na	1. T2DM vs. GDM<br>2. Onset Ramadan vs. After Ramadan	HbA1c, fructosamine	Average 25 week (Definition unclear)	na	Serum HbA1c reduced in GDM after Ramadan
Dikensoy (Turkey, 2009) [37]	Case-control study	Healthy pregnant women	65<br>1. Ramadan fasting: 36<br>2. No Ramadan fasting: 29	9/23/2006-10/23/2006	14.4 hours	1. Ramadan fasting: 23.4<br>2. No Ramadan fasting: 24.4<br>(Mean)	na	Fasting vs. Not fasting	Hematological parameter, weight gain during Ramadan, amniotic fluid index, Doppler flow indices, fetal weight, fetal biparietal diameter, fetal femur length, fetal biophysical profile	≥20 weeks of gestation	na	The results of this study showed that maternal serum cortisol level was elevated while LDL/HDL ratio was decreased in pregnant women, who were fasting during Ramadan. No untoward effect of Ramadan was observed on intrauterine fetal development.
Rakicioğlu (Turkey, 2006) [38]	Cross-sectional study	Healthy pregnant women	21	na	na	27.3<br>(Mean)	na	During Ramadan fasting vs. After Ramadan fasting	Breast milk composition	2-5 months after labor	na	Ramadan fasting had no significant effect on the macronutrient composition of the breast milk and consequently the growth of the infants. There were

												significant differences in some of the micronutrients such as zinc, magnesium, and potassium. The nutritional status of lactating women was affected by Ramadan fasting. All of the nutrient intakes (except vitamins A, E, and C) decreased during Ramadan.
Mirghani (United Arab Emirates, 2006) [39]	Case-control study	Healthy pregnant women	324<br>1. Ramadan fasting: 168<br>2. No Ramadan fasting: 156	11/6/2002-12/4/2002<br>10/29/2003-11/23/2003<br>10/15/2004-11/13/2004	13.9 hours	28.6	na	Fasting vs. Not fasting	Blood glucose, hematological parameter, GDM, hypertension, birth weight, gestational age at birth	≥30 weeks of gestation	Age, parity, and gestational age	Maternal diet restriction was associated with an increased risk of GDM and induction of labor. The frequency of neonatal admission to SCBU was increased.
Mirghani (United Arab Emirates, 2003) [40]	Cross-sectional study	Healthy pregnant women	162<br>1. Ramadan fasting: 81<br>2. No Ramadan fasting: 81	11/17/2001-12/12/2001	13.7 hours	1. Ramadan fasting: 29.1<br>2. No Ramadan fasting: 28.1<br>(Mean)	na	Fasting vs. Not fasting	Fetal breathing movement	≥30 weeks of gestation	Age, parity, and gestational age	Fetal breathing movement was reduced during maternal fasting.

Appearance, Pulse, Grimace, Activity, and Respiration (APGAR); Follicle-stimulating hormone (FSH); Gestational diabetes mellitus (GDM); Hemoglobin A1c (HbA1c); Hypertension (HTN); Intelligence Quotient (IQ); Luteinizing hormone (LH); Neutrophil gelatinase-associated lipocalin (NGAL); Neonatal intensive care unit (NICU); Oxidative stress index (OSI); Type 2 diabetes mellitus (T2DM); Total antioxidant status (TAS); Total oxidant status (TOS); Special Care Baby Unit (SCBU)

References

1. Parveen, R.; Khakwani, M.; Latif, M.; Tareen, A.U. Maternal and perinatal outcome after ramadan fasting. *Pakistan Journal of Medical Sciences* **2020**, *36*, 894.
2. Savitri, A.I.; Amelia, D.; Painter, R.C.; Baharuddin, M.; Roseboom, T.J.; Grobbee, D.E.; Uiterwaal, C.S. Ramadan during pregnancy and birth weight of newborns. *Journal of nutritional science* **2018**, *7*.
3. Safari, K.; Piro, T.J.; Ahmad, H.M. Perspectives and pregnancy outcomes of maternal Ramadan fasting in the second trimester of pregnancy. *BMC pregnancy and childbirth* **2019**, *19*, 1-10.
4. Engin-Ustun, Y.; Caglayan, E.K.; Kara, M.; Gocmen, A.Y.; Polat, M.F.; Aktulay, A. The effect of Ramadan fasting on sirtuin and visfatin levels. *Interventional Medicine and Applied Science* **2016**, *8*, 14-19.
5. AlMogbel, T.A.; Ross, G.; Wu, T.; Molyneaux, L.; Constantino, M.I.; McGill, M.; Harding, A.J.; Pech, C.; Alrasheed, A.A.; Wong, J. Ramadan and gestational diabetes: maternal and neonatal outcomes. *Acta diabetologica* **2022**, *59*, 21-30.
6. Hossain, N.; Samuel, M.; Mughal, S.; Shafique, K. Ramadan Fasting: Perception and maternal outcomes during Pregnancy. *Pakistan Journal of Medical Sciences* **2021**, *37*, 1262.
7. Gul, Z.; Rajar, S.; Shaikh, Z.F.; Shafique, K.; Hossain, N. Perinatal outcome among fasting and non fasting mothers during the month of Ramadan. *Pakistan Journal of Medical Sciences* **2018**, *34*, 989.
8. Sakar, M.; Balsak, D.; Verit, F.; Zebitay, A.; Buyuk, A.; Akay, E.; Turfan, M.; Demir, S.; Yayla, M. The effect of Ramadan fasting and maternal hypoalbuminaemia on neonatal anthropometric parameters and placental weight. *Journal of Obstetrics and Gynaecology* **2016**, *36*, 483-486.
9. Baynouna Al Ketbi, L.M.; Niglekerke, N.J.; Zein Al Deen, S.M.; Mirghani, H. Diet restriction in Ramadan and the effect of fasting on glucose levels in pregnancy. *BMC research notes* **2014**, *7*, 1-6.
10. Sakar, M.N.; Gultekin, H.; Demir, B.; Bakir, V.L.; Balsak, D.; Vuruskan, E.; Acar, H.; Yucel, O.; Yayla, M. Ramadan fasting and pregnancy: implications for fetal development in summer season. *Journal of Perinatal Medicine* **2015**, *43*, 319-323.
11. Abdelrahim, D.; Faris, M.E.; Hassanein, M.; Shakir, A.Z.; Yusuf, A.M.; Almeneessier, A.S.; BaHammam, A.S. Impact of Ramadan diurnal intermittent fasting on hypoglycemic events in patients with type 2 diabetes: a systematic review of randomized controlled trials and observational studies. *Frontiers in Endocrinology* **2021**, *12*, 624423.
12. Moradi, M. The effect of Ramadan fasting on fetal growth and Doppler indices of pregnancy. *Journal of research in medical sciences: the official journal of Isfahan University of Medical Sciences* **2011**, *16*, 165.

13. Abd-Allah Rezk, M.; Sayyed, T.; Abo-Elnasr, M.; Shawky, M.; Badr, H. Impact of maternal fasting on fetal well-being parameters and fetal–neonatal outcome: a case–control study. *The Journal of Maternal-Fetal & Neonatal Medicine* **2016**, *29*, 2834-2838.
14. Makvandi, S.; Karimi, L.; Mahdavian, M.; Bastami, A. No differences in hematological parameters of fasting and non-fasting pregnant women three months after Ramadan. *International Journal for Vitamin and Nutrition Research* **2019**.
15. Seckin, K.D.; Yeral, M.I.; Karslı, M.F.; Gultekin, I.B. Effect of maternal fasting for religious beliefs on fetal sonographic findings and neonatal outcomes. *International Journal of Gynecology & Obstetrics* **2014**, *126*, 123-125.
16. Savitri, A.I.; Yadegari, N.; Bakker, J.; van Ewijk, R.J.; Grobbee, D.E.; Painter, R.C.; Uiterwaal, C.S.; Roseboom, T.J. Ramadan fasting and newborn's birth weight in pregnant Muslim women in The Netherlands. *British Journal of Nutrition* **2014**, *112*, 1503-1509.
17. Awwad, J.; Usta, I.M.; Succar, J.; Musallam, K.; Ghazeeri, G.; Nassar, A. The effect of maternal fasting during Ramadan on preterm delivery: a prospective cohort study. *BJOG: An International Journal of Obstetrics & Gynaecology* **2012**, *119*, 1379-1386.
18. Gur, E.; Turan, G.; Ince, O.; Karadeniz, M.; Tatar, S.; Kasap, E.; Sahin, N.; Guclu, S. Effect of Ramadan fasting on metabolic markers, dietary intake and abdominal fat distribution in pregnancy. *Hippokratia* **2015**, *19*, 298.
19. Hızlı, D.; Yılmaz, S.S.; Onaran, Y.; Kafalı, H.; Danışman, N.; Mollamahmutoğlu, L. Impact of maternal fasting during Ramadan on fetal Doppler parameters, maternal lipid levels and neonatal outcomes. *The Journal of Maternal-Fetal & Neonatal Medicine* **2012**, *25*, 975-977.
20. Khoshdel, A.; Kheiri, S.; Hashemi-Dehkordi, E.; Nasiri, J.; Shabanian-Borujeni, S.; Saedi, E. The effect of Ramadan fasting on LH, FSH, oestrogen, progesterone and leptin in pregnant women. *Journal of Obstetrics and Gynaecology* **2014**, *34*, 634-638.
21. Ozturk, E.; Balat, O.; Ugur, M.G.; Yazıcıoglu, C.; Pence, S.; Erel, Ö.; Kul, S. Effect of Ramadan fasting on maternal oxidative stress during the second trimester: a preliminary study. *Journal of Obstetrics and Gynaecology Research* **2011**, *37*, 729-733.
22. Ziaee, V.; Kihanidoost, Z.; Younesian, M.; Akhavirad, M.-B.; Bateni, F.; Kazemianfar, Z.; Hantoushzadeh, S. The effect of Ramadan fasting on outcome of pregnancy. *Iranian Journal of Pediatrics* **2010**, *20*, 181.
23. Petherick, E.S.; Tuffnell, D.; Wright, J. Experiences and outcomes of maternal Ramadan fasting during pregnancy: results from a sub-cohort of the Born in Bradford birth cohort study. *BMC pregnancy and childbirth* **2014**, *14*, 1-9.
24. Kavehmanesh, Z.; Abolghasemi, H. Maternal Ramadan fasting and neonatal health. *Journal of perinatology* **2004**, *24*, 748-750.
25. Karateke, A.; Kaplanoglu, M.; Avci, F.; Kurt, R.K.; Baloglu, A. The effect of Ramadan fasting on fetal development. *Pakistan journal of medical sciences* **2015**, *31*, 1295.
26. Bayoglu Tekin, Y.; Guvendag Guven, E.S.; Mete Ural, U.; Yazici, Z.A.; Kirbas, A.; Kir Sahin, F. Evaluation of the effects of fasting associated

dehydration on maternal NGAL levels and fetal renal artery Doppler parameters. *The Journal of Maternal-Fetal & Neonatal Medicine* **2016**, *29*, 629-632.
27. Mirghani, H.; Weerasinghe, S.; Smith, J.; Ezimokhai, M. The effect of intermittent maternal fasting on human fetal breathing movements. *Journal of Obstetrics and gynaecology* **2004**, *24*, 635-637.
28. Afandi, B.O.; Hassanein, M.M.; Majd, L.M.; Nagelkerke, N.J. Impact of Ramadan fasting on glucose levels in women with gestational diabetes mellitus treated with diet alone or diet plus metformin: a continuous glucose monitoring study. *BMJ Open Diabetes Research and Care* **2017**, *5*, e000470.
29. Mirghani, H.M.; Weerasinghe, S.; Al-Awar, S.; Abdulla, L.; Ezimokhai, M. The effect of intermittent maternal fasting on computerized fetal heart tracing. *Journal of perinatology* **2005**, *25*, 90-92.
30. Malhotra, A.; Scott, P.; Scott, J.; Gee, H.; Wharton, B. Metabolic changes in Asian Muslim pregnant mothers observing the Ramadan fast in Britain. *British Journal of Nutrition* **1989**, *61*, 663-672.
31. Mirghani, H.M.; Salem, M.; Weerasinghe, S.D. Effect of maternal fasting on uterine arterial blood flow. *Journal of Obstetrics and Gynaecology Research* **2007**, *33*, 151-154.
32. Kiziltan, G.; Karabudak, E.; Tuncay, G.; Avsar, F.; Tuncay, P.; Mungan, O.; Meral, P. Dietary intake and nutritional status of Turkish pregnant women during Ramadan. *Saudi medical journal* **2005**, *26*, 1782-1787.
33. Khalaf, M.; Tammam, A.E.; Ibrahem, I.; Habib, D.M.; Abdellah, M.S.; Bahlol, M.; Khairy, M.; El Saman, A.M. Effect of Ramadan fasting on amniotic fluid index in last month of pregnancy. *Middle East Fertility Society Journal* **2015**, *20*, 54-56.
34. Azizi; Sadeghipour; Siahkolah; Rezaei-Ghaleh. Intellectual development of children born of mothers who fasted in Ramadan during pregnancy. *International journal for vitamin and nutrition research* **2004**, *74*, 374-380.
35. Kamyabi, Z.; Naderi, T. The effect of Ramadan fasting on amniotic fluid volume. *Saudi medical journal* **2004**, *25*, 45-46.
36. Ismail, N.A.M.; Raji, H.O.; Abd Wahab, N.; Mustafa, N.; Kamaruddin, N.A.; Jamil, M.A. Glycemic control among pregnant diabetic women on insulin who fasted during Ramadan. *Iranian journal of medical sciences* **2011**, *36*, 254.
37. Dikensoy, E.; Balat, O.; Cebesoy, B.; Ozkur, A.; Cicek, H.; Can, G. The effect of Ramadan fasting on maternal serum lipids, cortisol levels and fetal development. *Archives of gynecology and obstetrics* **2009**, *279*, 119-123.
38. Rakicioğlu, N.; Samur, G.; Topcu, A.; Topcu, A.A. The effect of Ramadan on maternal nutrition and composition of breast milk. *Pediatrics international* **2006**, *48*, 278-283.
39. Mirghani, H.M.; Hamud, O.A. The effect of maternal diet restriction on pregnancy outcome. *American journal of perinatology* **2006**, *23*, 021-024.
40. Mirghani, H.; Weerasinghe, D.; Ezimokhai, M.; Smith, J. The effect of maternal fasting on the fetal biophysical profile. *International Journal of Gynecology*

& Obstetrics **2003**, *81*, 17-21.
